# Transcriptome-Based Study on the Phylogeny and Hybridization of Marattialean Ferns (Marattiaceae)

**DOI:** 10.3390/plants12122237

**Published:** 2023-06-07

**Authors:** Jing Zhao, Xinmao Zhou, Shaoli Fang, Zhangming Zhu, Yuxin Li, Hong Yu, Zhaorong He

**Affiliations:** 1 School of Ecology and Environmental Science, Yunnan University, Kunming 650091, China; 2 School of Life Sciences, Yunnan University, East Outer Ring Road, Chenggong District, Kunming 650500, China

**Keywords:** *Angipteris sparsisora*, *Archangiopteris*, discordance, incomplete lineage sorting (ILS), hybridization, phylotranscriptomics

## Abstract

Marattiaceae is a phylogenetically isolated family of tropical eusporangiate ferns including six genera with more than one-hundred species. In Marattiaceae, monophyly of genera has been well-supported phylogenetically. However, the phylogenetic relationships among them were elusive and controversial. Here, a dataset of 26 transcriptomes (including 11 newly generated) were used to assess single-copy nuclear genes and to obtain the organelle gene sequences. Through phylotranscriptomic analysis, the phylogeny and hybridization events of Marattiaceae were explored and a robust phylogenomic framework for the evolution of Marattiaceae was provided. Using both concatenation- and coalescent-based phylogenies, the gene-tree discordance, incomplete lineage sorting (ILS) simulations, and network inference were examined. Except the low support with mitochondrial genes of Marattiaceae, nuclear genes and chloroplast genes strongly supported a sister relationship between Marattiaceae and leptosporangiate ferns. At the genus level, all phylogenetic analysis based on nuclear genes datasets recovered five genera in Marattiaceae as monophyletic with strong support. *Danaea* and *Ptisana* were the first two diverged clades in turn. *Christensenia* was a sister clade to the clade *Marattia* + *Angiopteris* s.l. In *Angiopteris* s.l., three clades (*Angiopteris* s.s., the *Archangiopteris* group, and *An. sparsisora*) were well identified with maximum support. The *Archangiopteris* group was derived from *Angiopteris* s.s. at ca. 18 Ma. The putative hybrid species *An. sparsisora* between *Angiopteris* s.s. and the *Archangiopteris* group was verified by the species network analyses and the maternal plastid genes. This study will improve our understanding for using the phylotranscriptomic method to explore phylogeny and investigate hybridization events for difficult taxa in ferns.

## 1. Introduction

Marattiales Link is one of the most ancient lineages of ferns [1]. Only one family, Marattiaceae Kaulf., was recognized in extant Marattiales, including six genera with ca. 110 species [2]. Marattiales have one of the richest fossil records of all fern lineages (e.g., [3,4,5,6,7,8,9]). Although extant Marattiales are essentially restricted to the tropical regions of the world [1,10], their fossil records have been found in all continents [9,11,12,13,14,15,16,17,18], making it an ideal group for divergence time estimation and learning about the process driving evolution and diversification [19,20]. The eusporangiate Marattiales were often resolved as the sister group of the leptosporangiate ferns (=Polypodiidae Cronquist, Takht. & W. Zimm.) [19,21,22,23,24,25,26,27,28,29,30,31,32,33,34,35,36,37]. However, some studies instead resolved Marattiaceae as sister to the clade Psilotales Prant + Ophioglossales Link [38,39,40] or to Equisetales DC. ex Bercht. & J. Presl [41].

**Figure 1:**
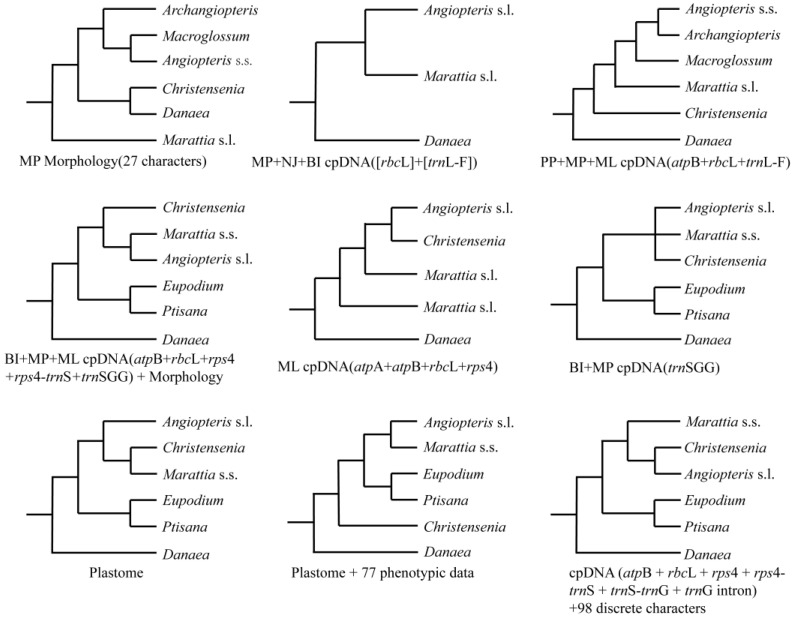
Summary of the topologies among Marattiales in previous studies.

In Marattiaceae, Murdock [1] recognized six genera based on a phylogenetic relationship and morphological characters [1,10]. He divided *Marattia* s.l. into three genera (*Marattia* s.s. Sw., *Eupodium* J.Sm., and *Ptisana* Murdock) and combined another three genera (*Angiopteris* Hoffm. s.s., *Archangiopteris* Christ & Giesenh, and *Macroglossum* Copel.) into *Angiopteris* s.l. Murdock’s [10] classification was widely accepted and adopted by subsequent scholars [2,42,43,44]. In all previous phylogenetic studies for Marattiaceae, *Danaea* was recovered as sister to the rest of Marattiaceae and the clade *Eupodium* + *Ptisana* was always well-supported (Figure 1). However, depending on different datasets and analysis methods, phylogenetic relationships among *Angiopteris* s.l., *Christensenia*, *Marattia*, and the clade *Eupodium* + *Ptisana* varied (Figure 1; also see [10,19,20,21,34,45,46,47,48,49]). Recently, by incorporating good sampling and abundant molecular and morphological data for the extant species, Lehtonen et al. [19] reconstructed the phylogeny of Marattiaceae. Thus, *Christensenia* was resolved as the sister group to *Marattia*. May et al. [20] inferred a similar phylogeny to that of Lehtonen et al. [19], but *Christensenia* was resolved as sister to *Angiopteris* s.l. instead of to *Marattia*.

Although Angiopteridaceae Fée ex J was reduced into Marattiaceae and widely accepted and recognized, Angiopteridaceae (=*Angiopteris* s.l.) was a historically accepted and adopted family, especially in Chinese flora and works (e.g., [50,51,52,53]), which often include three genera: *Angiopteris* s.s., *Archangiopteris*, and *Macroglossum* [54]. *Angiopteris* s.l. (=Angiopteridaceae Fée ex J) includes three historic genera: *Angiopteris* s.s., *Archangiopteris* (=the *Archangiopteris* group), and *Macroglossum* [54,55]. *Angiopteris* s.l. has 30~40 species and represents the highest diversity of Marattiaceae in Paleotropics, especially in China, with ca. 30 species [2,42]. Although *Angiopteris* s.l. has always been resolved as a well-supported monophyletic and morphologically distinguishable group, its phylogenetic placement was uncertain in Marattiaceae [10,47,56]. In *Angiopteris* s.l., the *Archangiopteris* group contains about 10 species and is mainly distributed in Southwest China and northern Vietnam [51,52,53]. Morphologically, species of the *Archangiopteris* group are smaller, have simple pinnate leaves (except *An. bipinnata* (Ching) J. M. Camus = (*Ar. bipinnata* (Ching) J. M. Camus), suberect or creeping rhizomes, one pulvinus (rarely reached five) on the stipe, long and thin sori, and spiny spores, which distinguished them from *Angiopteris* s.s. However, some hypotheses about natural hybrid species with transitional morphology among *Angiopteris* s.s. and the *Archangiopteris* group were also reported, e.g., *An. sparsisora* Ching and *An. sugongii* Gui L. Zhang, J.Y. Xiang & Ting Wang [42,51,57,58,59]. A triploid species (*An. itoi* (W. C. Shieh) J. M. Camus (=*Ar. itoi* J. M. Camus)) was reported as a hybrid between *An. lygodiifolia* Rosenstock and *An. somae* (Hayata) Makino and Nemoto (=*Ar. somae* Hayata) [60], which was supported by the RAPD markers [60] and chromosome numbers [61]. However, these hybrids have never been studied and tested in phylogenetic analysis. The morphological evolution between *Angiopteris* s.s. and the *Archangiopteris* group is another historically debated question since *Archangiopteris* was reconstructed. Some scholars insisted that the *Archangiopteris* group has primitive morphological characteristics [47,62], which is supported by some earlier molecular studies (e.g., [23,56,63]). However, comparative study on morphology shows that the *Archangiopteris* group is very likely divergent from *Angiopteris* s.s. [51,64]. The *Archangiopteris* group has been always resolved as a monophyletic group in phylogeny but clustered within *Angiopteris* s.s. [10]. However, in previous studies, only a few species of the *Archangiopteris* group were sampled and the phylogenetic relationship between the *Archangiopteris* group and *Angiopteris* s.s. was poorly resolved.

One of the underlying reasons for this controversy over the phylogeny in Marattiaceae is due to the limited molecular data. Nowadays, phylogenetic inference based on RNA-Seq is more efficient and cost-effective when lacking whole genome data [39], in which sequences from the nuclear genome contain the majority of genetic information among the three genomes in plants [34]. RNA-seq data have been used to explore the backbone phylogeny in ferns [28,34,37,39,40], but such data were rarely used for specific families or genera in ferns before. In this manuscript, using RNA-seq data, a large number of nuclear genes can be used either for inferring the phylogenetic relationships or dealing with hybridization, speciation, and incomplete lineage sorting in closely related species of Marattiaceae, as demonstrated in previous studies in angiosperms [65,66,67,68].

To explore the deeper relationships of Marattiales, one set of broad representative taxon data, including newly generated transcriptomes data, has been used in this study. Multiple strategies, including different datasets and detection and removal of intra-locus recombinant regions, were applied to minimize systematic errors. Moreover, concatenated approaches, coalescent methods, network analysis, and incomplete lineage sorting (ILS) simulation were used to provide new insights into the evolution of Marattiales.

In this study, we used twenty-six transcriptomes, including Marattiaceae (nearly all extant genera, sixteen samples), Ophioglossaceae (two samples), Psilotaceae (one sample), Polypodiidae (two samples), Equisetaceae (one sample), and (Amborellaceae) in order to (1) revisit the phylogenetic framework of Marattiaceae; (2) investigate phylogenetic incongruence among nuclear gene trees based on different datasets and analysis methods using genome-scale data; (3) explore the phylogenetic relationship between *Angiopteris* s.s. and the *Archangiopteris* group; and (4) test the hybridization events of an assumed hybrid species, *An. sparsisora*, between *Angiopteris* s.s. and the *Archangiopteris* groups.

## 2. Results

### 2.1. Characteristics of Transcriptomes and Nuclear Gene Datasets

We used sixteen transcriptomes (including eleven newly generated in this study) with a wide range of Marattiaceae, including fourteen species from five genera, and ten species from six families (Equisetaceae, Psilotaceae, Ophioglossaceae, Osmundaceae Martinov, Hymenophyllaceae Mart., and Amborellaceae Pichonas) were used as outgroups in this study (Table 1). Of the 11 newly generated transcriptomes, we finally retained a total of 41,613,264–52,013,624 reads of the clean data. After data quality filtering, the percentage of GC content ranged from 46.78% to 47.70% (Table S3).

Twenty single-copy nuclear gene datasets were generated for phylogenetic analysis with concatenation and coalescent approaches. Including all twenty-six samples generated by Orothofinder(O) and SonicParanoid (S), four datasets comprising the aligned amino acid sequences (aa: 26-O-aa and 26-S-aa) and the codon-aligned nucleotide sequences (cds: 26-O-cd and 26-S-cds) were used. Four datasets (16-O-aa, 16-O-cds, 16-S-aa, and 16-S-cds) including only sixteen samples from the Marattiaceae were used to test the earliest evolutionary lineage in Marattiaceae. Due to computational restrictions and given our main focus on potential reticulation among major clades of Marattiaceae at family level, genus level, and species level, another 12 datasets (Family-O-aa, Family-O-cds, Family-S-aa, Family-S-cds, Genus-O-aa, Genus-O-cds, Genus-S-aa, Genus-S-cds, Species-O-aa, Species-O-cds, Species-S-aa, and Species-S-cds) were generated. Statistics of the datasets are presented in Table 2. With the exception of percentage of the pairwise identity and identical sites, all the remaining statistics in datasets generated by SonicParanoid are generally 1.1–2.8 times higher than those of datasets generated by OrthoFinder.

### 2.2. Phylogenetic Reconstruction Using Single-Copy Nuclear Genes

**Figure 2:**
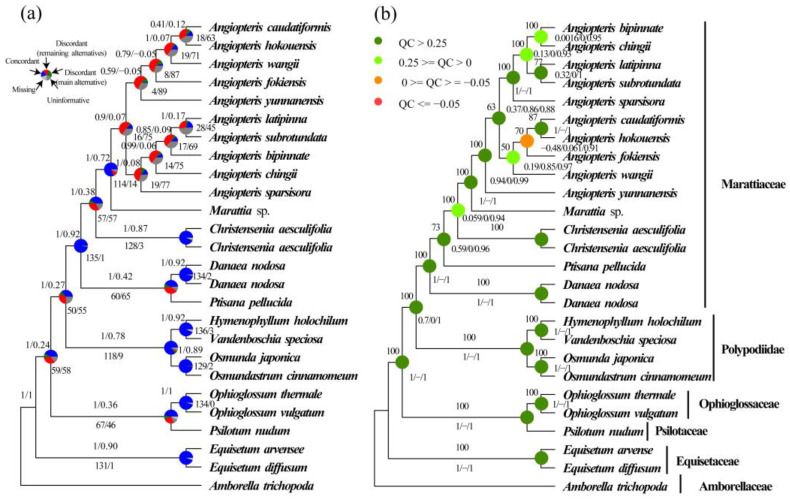
Phylogenetic analysis of species trees based on 26-S-aa in Marattiaceae. (**a**). Astral-II posterior probabilities (AS-PP) and internode certainty all (ICA) values are shown above branches, and numbers of gene trees (concordant/conflicting) are shown below branches. Pie charts present the proportion of congruent gene trees that supports that clade (blue), the proportion of discordant gene trees of the main alternative topology for that clade (green), the proportion of discordant trees for the remaining alternative topologies (red), the proportion of uninformative gene trees (dark gray; bootstrap support < 50%), and the proportion of missing data (light gray). (**b**). Maximum likelihood bootstrap support (ML-BS) is shown above the branches, and quartet sampling internal node scores (quartet concordance/quartet differential/quartet informativeness) are shown below branches.

**Figure 3:**
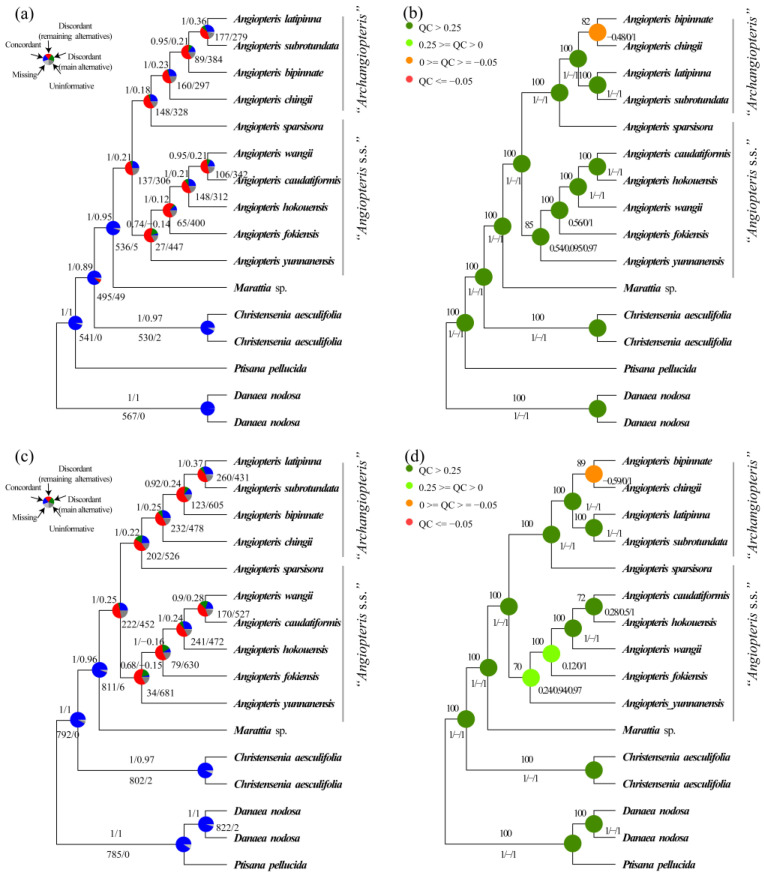
Phylogenomic analysis of species trees based on 16-O-cds and 16-S-cds datasets in Marattiaceae ((**a**,**b**). 16-O-cds datasets, (**c**,**d**). 16-S-cds datasets). (**a**,**c**). Astral-II posterior probabilities (AS-PP) and internode certainty all (ICA) values are shown above branches, and numbers of gene trees (concordant/conflicting) were shown below branches. Pie charts present the proportion of congruent gene trees that supports that clade (blue), the proportion of discordant gene trees of the main alternative topology for that clade (green), the proportion of discordant trees for the remaining alternative topologies (red), the proportion of uninformative gene trees (dark gray; bootstrap support <50%), and the proportion of missing data (light gray). (**b**,**d**). Maximum likelihood bootstrap support (ML-BS) is shown above the branches, and quartet sampling internal node scores (quartet concordance/quartet differential/quartet informativeness) are shown below branches.

**Figure 4:**
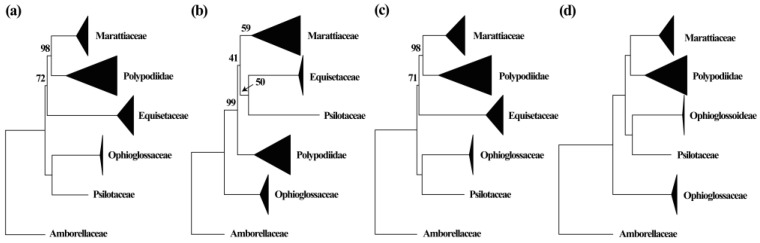
Simplified phylogeny of Marattiaceae based on different datasets. (**a**). Maximum likelihood phylogeny based on CT dataset. (**b**). Maximum likelihood phylogeny based on MT dataset. (**c**). Maximum likelihood phylogeny based on the combined dataset of CM. (**d**). Consensus of 40 species trees from all inferences with IQ-tree and Astral-II using single-copy nuclear genes. The sizes of hollow triangles are in proportion to the sampled sizes of individual clades. Support values are shown along the branches; values of 100 and 1 are not displayed.

**Figure 5:**
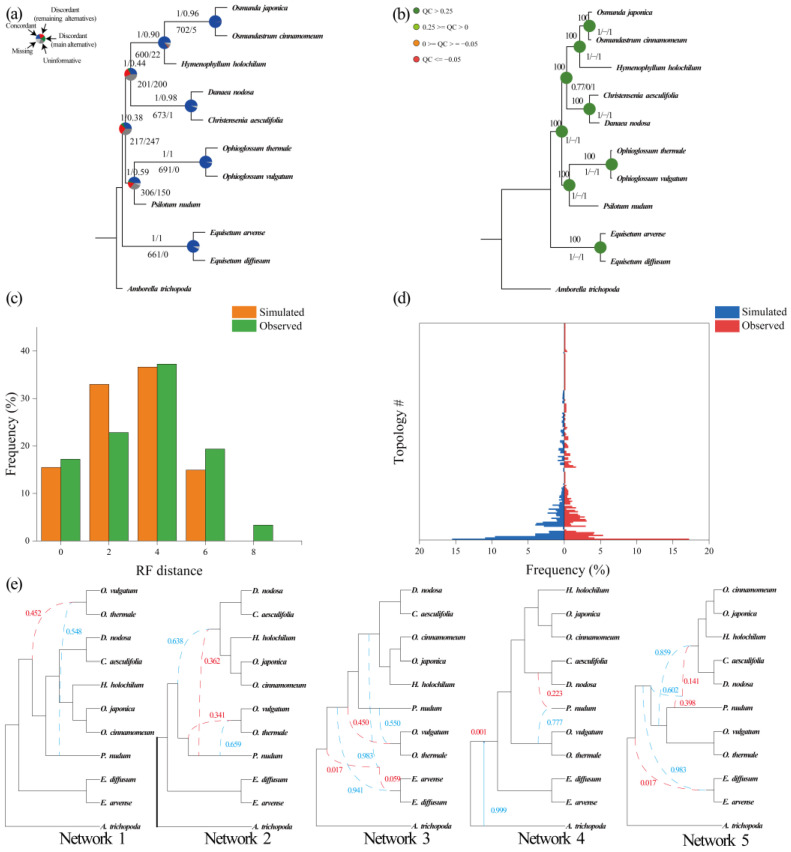
(**a**,**b**). Phylogenetic analysis of species tree with Family-O-aa dataset in Marattiaceae ((**a**). Astral-II posterior probabilities (AS-PP) and internode certainty all (ICA) scores are shown above branches and numbers of gene trees (concordant/conflicting) are shown next to the nodes; (**b**) maximum likelihood bootstrap support (ML-BS) is shown above the branches. Quartet sampling internal node scores (quartet concordance/quartet differential/quartet informativeness) are shown below branches); (**c**) distributions of topology frequencies of observed and simulated gene trees based on Family-O-aa dataset; (**d**) distribution of Robinson-Foulds distances of the simulated and observed gene trees to the coalescent tree; (**e**) species network inferred from PhyloNet pseudolikelihood analyses with one to five hybridization events based on Family-O-aa dataset. Red and blue curved branches indicate the minor and major edges of hybrid nodes, respectively. Numbers next to curved branches indicate inheritance probabilities for each hybrid node.

**Figure 6:**
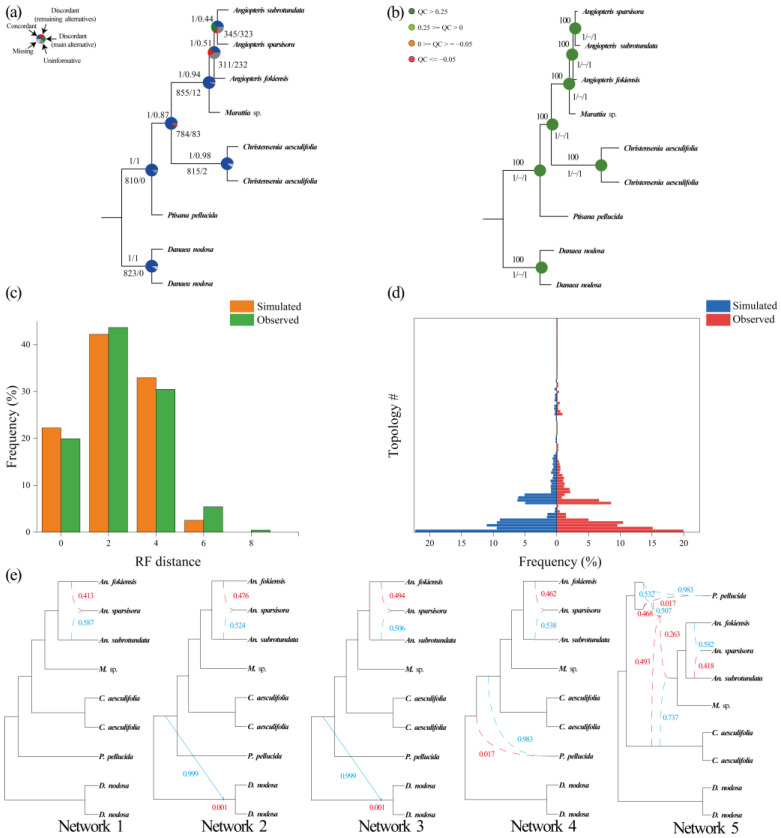
(**a**,**b**). Phylogenetic analysis of species tree with Genus-O-cds dataset in Marattiaceae ((**a**). Astral-II posterior probabilities (AS-PP) and internode certainty all (ICA) scores are shown above branches and numbers of gene trees (concordant/conflicting) are shown next to the nodes; (**b**) maximum likelihood bootstrap support (ML-BS) is shown above the branches. Quartet sampling internal node scores (quartet concordance/quartet differential/quartet informativeness) are shown below branches); (**c**) distributions of topology frequencies of observed and simulated gene trees based on Genus-O-cds dataset; (**d**) distribution of Robinson-Foulds distances of the simulated and observed gene trees to the coalescent tree; (**e**) species network inferred from PhyloNet pseudolikelihood analyses with one to five hybridization events based on Genus-O-cds dataset. Red and blue curved branches indicate the minor and major edges of hybrid nodes, respectively. Numbers next to curved branches indicate inheritance probabilities for each hybrid node.

**Figure 7:**
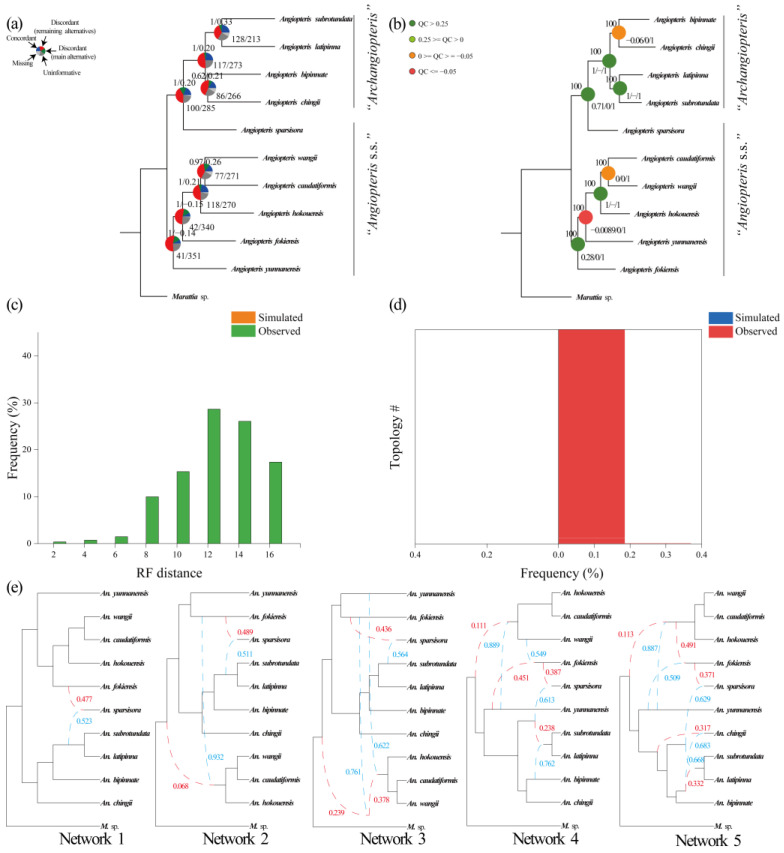
(**a**,**b**). Phylogenetic analysis of species tree with Species-O-aa dataset in Marattiaceae ((**a**). Astral-II posterior probabilities (AS-PP) and internode certainty all (ICA) scores are shown above branches and numbers of gene trees (concordant/conflicting) are shown next to the nodes; (**b**) maximum likelihood bootstrap support (ML-BS) is shown above the branches. Quartet sampling internal node scores (quartet concordance/quartet differential/quartet informativeness) are shown below branches); (**c**) distributions of topology frequencies of observed and simulated gene trees based on Species-O-aa dataset; (**d**) distribution of Robinson-Foulds distances of the simulated and observed gene trees to the coalescent tree; (**e**) species network inferred from PhyloNet pseudolikelihood analyses with one to five hybridization events based on Species-O-aa dataset. Red and blue curved branches indicate the minor and major edges of hybrid nodes, respectively. Numbers next to curved branches indicate inheritance probabilities for each hybrid node.

Based on the 20 single-copy nuclear gene datasets, a total of 40 species trees were inferred using concatenation and coalescent approaches in IQ-tree (Figure 2b, Figure 3b,d, Figure 4d, Figure 5b, Figure 6b, Figure 7b, Figures S1b,d, S2b, S3–S5b, S6b,d and S7–S12b) and Astral-II (Figure 2a, Figure 3a,c, Figure 4d, Figure 5a, Figure 6a, Figure 7a, Figures S1a,c, S2a, S3–S5a, S6a,c and S7–S12a), respectively. In analyses of nucleotide and amino acid sequences, Marattiaceae were found to be sister to Polypodiidae (ML-BS = 100; AS-PP = 1; Figure 2a,b, Figure 4d, Figure 5a,b, Figures S1a–d, S2a,b and S3–S5a,b). The conflict analyses confirmed the monophyly of Marattiaceae and placed it as sister to Polypodiidae by a majority of gene trees, 50 (out of 139, internode certainty all (ICA) = 0.27) (Figure 2a), 201 (out of 712, ICA = 0.44) (Figure 5a), 24 (out of 61, ICA = 0.21) (Figure S1a), 33 (out of 77, ICA = 0.28) (Figure S1c), 63 (out of 154, ICA = 0.33) (Figure S2a), 298 (out of 793, ICA = 0.56) (Figure S3a), 409 (out of 1234, ICA = 0.52) (Figure S4a), 519 (out of 1329, ICA = 0.70) (Figure S5a), and having high quartet sampling (QS) support (0.45–0.77/0/0.98–1) (Figure 2b, Figure 5b, Figures S1b,d, S2b and S3–S5b).

At the genus level in Marattiaceae, in those datasets (26-O-aa/26-O-cds/26-S-aa/26-S-cds), except that the ML tree of 26-S-aa resolved *Danaea* as the first evolutionary lineage (Figure 2b), the remaining species trees resolved the *Danaea* + *Ptisana* clade as the first evolutionary lineage (Figure 2a,b, Figures S1a–d and S2a,b). However, the former (*Danaea* as the first evolutionary lineage) was with full QS support (1/−/1) (Figure 2b), the latter (the *Danaea* + *Ptisana* clade as the first evolutionary lineage *a*) with counter-support QS values (−0.38–0.2/0/0.94–0.99) (Figures S1b,d and S2b). To further detect the first diverging lineage in Marattiaceae, the 16 taxa datasets (16-O-aa/16-O-cds/16-S-aa/16-S-cds) using *Danaea* (ML-BS = 100, AS-PP = 1; Figure 3a,b and Figure S6a,b) or *Danaea* + *Ptisana* (ML-BS = 100, AS-PP = 1; Figure 3c,d and Figure S6c,d) as outgroups were analyzed, respectively. The conflict analyses of both have full QS support (1/−/1) (Figure 3b,d and Figure S6b,d) but with higher gene trees support in these trees using *Danaea* as the first divergent clade by 99% (567 out of 571, ICA = 1) (Figure 3a) and 98% (453 out of 460, ICA = 1) (Figure S6a) than the trees using *Danaea* and *Ptisana* 91% (785 out of 863, ICA = 1) (Figure 3c), and 88% (548 out of 626, ICA = 1) (Figure S6c). We presume that *Danaea* was the first evolutionary lineage in Marattiaceae (for more discussion, see “Discussion” section). In all subsequent analyses, *Danaea* was always rooted as the outgroup.

In our phylogenetic analyses based on the datasets of 16-O-cds, 16-S-cds, 16-O-aa, 16-S-aa, Genus-O-cds, Genus-S-cds, Genus-O-aa, and Genus-S-aa, the monophyly and phylogenetic relationships of five genera in Marattiaceae were well-resolved (ML-BS = 100; AS-PP = 0.98–1; Figure 3a,b, Figure 6a,b, Figures S6a,b and S7–S9a,b). *Danaea* was rooted as an outgroup. *Ptisana* is sister to the rest of Marattiaceae. *Angiopteris* s.l. is sister to *Marattia*, and they are together sister to *Christensenia*. In *Angiopteris* s.l., three well-supported clades were identified: *Angiopteris* s.s., the *Archangiopteris* group, and *An. sparsisora* (a potential hybrid; see below).

Analysis of phylogenetic conflicts confirmed a high level of gene tree accordance at genus level and monophyly of *Angiopteris* s.l., *Danaea*, *Ptisana*, *Christensenia*, and *Marattia* with full QS support values (1/−/1) (Figure 2a,b, Figure 3a–d, Figures S1a–d, S2, S3a,b and S5a,b). *Danaea* is sister to other genus in Marattiaceae by 567 (out of 571; ICA = 1) in 16-O-cds datasets (Figure 3a), 823 (out of 910; ICA = 1) in Genus-O-cds datasets (Figure 6a), 453 (out of 460; ICA = 1) in 16-O-cds datasets (Figure S6a), 630 (out of 699; ICA = 1) in Genus-O-aa datasets (Figure S7a), 989 (out of 1101; ICA = 1) in Genus-S-aa datasets (Figure S8a), and 1254 gene trees (out of 1387; ICA = 1) in Genus-S-cds datasets (Figure S9a), respectively. *Ptisana* as the sister to the clade *Christensenia* + *Marattia* + *Angiopteris* s.l. was highly supported by 541 (out of 571, ICA = 1) (Figure 3a), 810 (out of 910; ICA = 1) (Figure 6a), 431 (out of 460; ICA = 1) (Figure S6a), 618 (out of 699; ICA = 1) (Figure S7a), 965 (out of 1101; ICA = 1) (Figure S8a), and 1232 gene trees (out of 1387; ICA = 1) (Figure S9a), respectively. The sister relationship between *Christensenia* and the clade *Marattia* + *Angiopteris* s.l. was supported by the majority of gene trees, 495 (out of 571; ICA = 0.89) (Figure 3a), 784 (out of 910; ICA = 0.87) (Figure 6a), 341 (out of 460; ICA = 0.84) (Figure S6a), 530 (out of 699; ICA = 0.82) (Figure S7a), 836 (out of 1101; ICA = 0.84) (Figure S8a), and 1206 (out of 1387; ICA = 0.89) (Figure S9a), respectively. *Marattia* was sister to *Angiopteris* s.l., supported by 536 (out of 571, ICA = 0.95) (Figure 3a), 855 (out of 912, ICA = 0.94) (Figure 6a), 412 (out of 460, ICA = 0.87) (Figure S6a), 609 (out of 699, ICA = 0.89) (Figure S7a), 948 (out of 1101, ICA = 0.90) (Figure S8a), and 1305 gene trees (out of 1387, ICA = 0.95) (Figure S9a).

In *Angiopteris* s.l., interspecific relationships of *Angiopteris* s.s. and the *Archangiopteris* group were resolved with medium to high support (ML-BS = 60–100; AS-PP = 0.49–1; QS = −0.54–1/0–0.75/0.96–1; ICA = −0.15–0.42) (Figure 3a,b, Figure 7a,b, Figures S6a,b and S10–S12a,b). *Angiopteris* s.s. (except *An. sparsisora*) and the *Archangiopteris* group were recovered as monophyletic in all concatenation and coalescent species trees, with support by only 87/137 gene trees (out of 460/571; ICA = 0.16–1) (Figure 3a,b, Figure 7a,b, Figures S6a,b and S10–S12a,b) but with strong support (ML-BS = 100; AS-PP = 0.98–1) and full QS support (1/−/1) (Figure 3a,b, Figure 7a,b, Figures S6a,b and S10–S12a,b).

### 2.3. Phylogenetic Reconstruction of Organelle Genes

Using the customized pipeline, we recovered 87 and 32 protein-coding genes from the CT and MT datasets, respectively. Finally, alignments of CT dataset with 78,897 bp in length, MT dataset with 36,568 bp in length, and the combined matrix (CM) with 115,465 bp in length were used to infer the phylogeny. Detailed information on the assembled chloroplast genes and mitochondria genes are provided in Figshare: https://doi.org/10.6084/m9.figshare.21707546.

At the family level, phylogenetic analysis of the CT and CM datasets recovered Marattiaceae sister to Polypodiidae with strong support (ML-BS = 98; Figure 4a,c, Figures S13 and S15), but the MT dataset resolved Marattiaceae as sister to the clade Equisetaceae + Psilotaceae with weak support (ML-BS = 41; Figure 4b and Figure S14). At the genus level, the plastid genes result corroborates the nuclear phylogeny (Figure S13). The phylogeny based on mitochondrion genes (MT dataset) inferred that five genera are non-monophyletic but with extremely weak support (Figure S14). The CM dataset infers that the clade *Danaea + Ptisana* is the first diverged clade with strong support (ML-BS = 90). *Marattia* is nested with *Angiopteris* s.l., and they are together sister to *Christensenia* (Figure S15).

To avoid the impact of missing data of chloroplast genes, we only retain *Marattia* sp. as the outgroup to infer phylogeny of *Angiopteris* s.l. with chloroplast genes. Three medium-supported clades (*Angiopteris* s.s. (ML-BS = 97), *Angiopteris sparsisora* (a putative hybrid species) clade, and the *Archangiopteris* group (ML-BS = 51)) were identified (Figure S16). *Angiopteris sparsisora* was sister to *Angiopteris* s.s. based on chloroplast phylogenomics instead of sister to the *Archangiopteris* group in nuclear genes.

### 2.4. Coalescent Simulations

To investigate whether ILS alone could explain the cytonuclear and nuclear gene-tree discordance, coalescent simulation analysis with datasets of Family-O-aa/Family-O-cds/Family-S-aa/Family-S-cds/Genus-O-aa/Genus-O-cds/Genus-S-aa/Genus-S-cds/Species-O-aa/Species-O-cds/Species-S-aa/Species-S-cds were executed. The datasets of Family-O-aa/Family-O-cds/Family-S-aa/Family-S-cds mainly focused on the ILS at the family level in Marattiaceae, the datasets of/Genus-O-aa/Genus-O-cds/Genus-S-aa/Genus-S-cds mainly focused on the ILS at the genus level in Marattiaceae, and the datasets of Species-O-aa/Species-O-cds/Species-S-aa/Species-S-cds mainly focused on the ILS in *Angiopteris* s.l.

At the genus level and family level, distribution of tree-to-tree distances between simulated gene trees and the observed gene trees largely overlapped (Figure 5, Figure 6c,d, Figures S3–S5c,d and S7–S9c,d) using the Family-O-aa/Family-O-cds/Family-S-aa/Family-S-cds/Genus-O-aa/Genus-O-cds/Genus-S-aa/Genus-S-cds datasets. However, using the same ILS simulation workflows within the remaining datasets at species level, ILS tests for most of the discordance in *Angiopteris* s.l. were accounted for. All the analyses distinguishing between simulated and observed trees distance distributions show that ILS cannot account for the discordance in *Angiopteris* s.l. (Figure 7c,d and Figures S10–S12c,d).

### 2.5. Network Analysis

The datasets used in ILS simulations were used to perform phylogenetic network analysis in PhyloNet while accounting for both hybridization and ILS. At the family level (Figure 5e and Figures S3–S5e), the inferred Network4 has the highest log pseudo-likelihood (Table 3). Gene flow was detected in all outgroups (Figure 5e and Figures S3–S5e), indicating that hybridization could be a possible cause of discordance. Up to five hybridization events at the generic level in Marattiaceae were also examined; the inferred network with two reticulation events has the highest log pseudo-likelihood (Table 3). One reticulation event from ancestral *Angiopteris fokiensis* and *An. Subrotundata* lineage to *An. Sparsisora* was inferred in all five examinations (Figure 6e), with an inheritance probability of 0.413–0.582 from the *An. fokiensis* lineage and 0.418–0.587 from the *An. subrotundata* lineage, and it was detected in all five examinations in the Genus-S-cds dataset (Figure S9e), network1-network3 in the Genus-O-aa dataset (Figure S7e), and network2-network5 in the Genus-S-aa dataset (Figure S8e). Three (Figure S7e: Network 3, Figure S8e: Network 4), six (Figure 6e: Network 2-Network3, Figure S9e: Network 2−Network 5), and three (Figure 6e: Network 4−Network5; Figure S8e: Network1) inferred hybridization events between *Christensenia*, *Danaea*, and *Ptisana* were also detected. For the reticulation event of *Christensenia*, *Danaea*, and *Ptisana*, one of the gene flow events was basically from ancestral in Marattiaceae (except *Danaea*) lineage to the ancestors of each other (Figure 6e: Network 2−Network5; Figure S7e: Network3; Figure S8e: Network1 and Network4; Figure S9e: Network2−Network5).

In *Angiopteris* s.l., up to five hybridization events among *Angiopteris* s.s. were allowed (Figure 7e and Figures S10–S12e). *Angiopteris sparsisora* was inferred to have a genetic contribution from the lineage of *Angiopteris* s.s. and the *Archangiopteris* group in all datasets, and its reticulation event was detected in all 20 examinations. *Angiopteris sparsisora* has an inheritance probability of 0.371–0.506 from the *Angiopteris* s.s. lineage and 0.494–0.629 from the *Archangiopteris* group lineage (Figure 7e and Figures S10–S12e). Although the inferred network with the highest log pseudo-likelihood included one reticulation event (Table 3, except Species-S-cds dataset with two reticulations: −267.86003727524945), the genera-level hybridization events that included more reticulations, which were mainly caused by hybridization events among *Angiopteris*, were calculated (Figure 6e and Figures S7–S9e).

### 2.6. Divergence Dating Estimation

**Figure 8:**
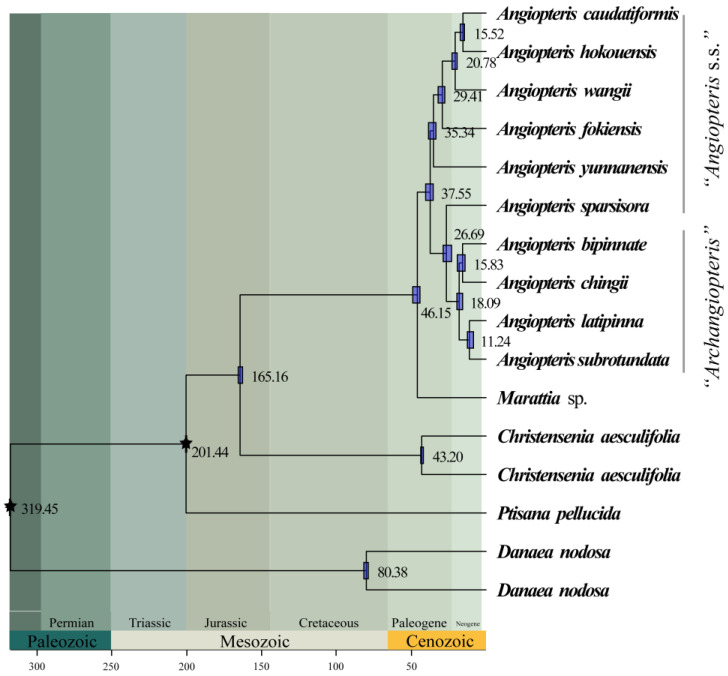
Time-calibrated phylogeny of Marattiaceae. The times were inferred by treePL based on 16-O-cds dataset. The divergence times are shown behind the nodes, the 95% highest posterior densities are represented as blue bars, and branch length are proportional to relative ages (in millions of years; see scale at bottom of tree).

Our analyses infer a stem age of Marattiaceae dating to the late Carboniferous–early Permian (ca. 319 Ma; Figure 8). The split between *Danaea* and the rest of extant Marattiaceae occurred in ca. 201 Ma, corresponding to the late Triassic. The stem age of *Christensenia* was estimated to be ca. 165 Ma (middle Jurassic), and the crown age was ca. 43 Ma (Figure 8). The stem age of *Marattia* was estimated to be ca. 46 Ma (middle Paleogene; Figure 8). In *Angiopteris* s.l., the *Archangiopteris* group diverged from *Angiopteris* s.s. at ca. 37 Ma during middle Cenozoic (Figure 8).

## 3. Discussion

### 3.1. New Insights into Phylogeny of Marattiaceae

In previous phylogenetic reconstruction for Marattiaceae, less than four genera and four species were used for phylogenetic analysis based on nuclear genes [28,34,37,38,39,40]. In this work, using a broad taxon sample of newly generated transcriptomes, the phylogenetic relationships of Marattiales were explored through analysis of uniparentally inherited organelle genes and single-copy biparental nuclear genes.

In this study, two alternative relationships of Marattiaceae with other fern lineages were inferred: sister to Polypodiidae or sister to the clade Equisetaceae + Psilotaceae. All the phylogenies inferred from datasets of nuclear, plastid, or concatenated plastid and mitochondrial genes supported the sister relationship between Marattiaceae and Polypodiidae (Figure 4a,c,d), which is consistent with previous results based on plastid markers [19,21,22,23,24,25,26,30,31,32,33,35], concatenated plastid and mitochondrial genes [27], and nuclear genes [28,34]. However, our mitochondrial dataset resolved Marattiaceae as sister to the clade Equisetaceae + Psilotaceae with weak support (Figure 4b). As Murdock [10] and Lehtonen et al. [19] have noted, inferences of the first diverging lineage in extant Marattiaceae are sensitive to datasets choice, LBA, outgroup choice, and use of optimality criteria.

With extensive outgroups, two alternatives for the first divergent lineage, *Danaea* (Figure 2b) or the clade *Danaea* + *Ptisana* (Figure 2a, Figures S1a–d and S2a,b), were found. The latter, however, has a higher proportion of gene tree conflicts and low ICA and QS values (Figure 2a,b, Figures S1a–d and S2a,b). To further test the first lineage of Marattiaceae, conflict analysis results of different root settings were compared using single-copy nuclear genes specific to Marattiaceae, *Danaea* as the first evolutionary lineage of Marattiaceae with higher gene proportion (Figure 3a–d and Figure S6a–d). In addition, phylogenetic reconstruction based on chloroplast genes also shows the root of Marattiaceae separates *Danaea* from the rest of the genus (Figure S13). In all, our phylogenomic results are consistent with Murdock’s [10] study based on chloroplast DNA data but with higher support for each clade (Figure 3a,b, Figure 6a,b, Figures S6a,b and S7–S9a,b). Although *Eupodium* was not included in our phylogenetic analysis, it has been resolved as the sister to *Ptisana* in all previous studies (e.g., [1,10,19,20,48]). Here, TreeShrink was further selected to reduce the influence of long branch attraction and improved the accuracy of phylogeny in all datasets, which could identify and remove those sequences that lead to unrealistically long branch lengths within each cluster [70]. Nearly all previous phylogenetic studies have resolved *Danaea* as the first divergent clade in Marattiaceae [1,10,19,20,23,48,56,63]. In this study, we also recovered *Danaea* as a sister to the rest of the family in phylogenetic analyses of the multi-locus plastid genes; all the conflict analyses based on different single-copy nuclear gene datasets and the all analyses were concordant (ICA = 1; QS = 1/−/1) (Figure 3a,b, Figure 6a,b, Figures S6a,b, S7–S9a,b and S13). *Ptisana* was sister to the clade comprised by three genera (*Angiopteris* s.l., *Christensenia*, and *Marattia*) in Marattiaceae. In some previous studies, phylogenetic placement of *Danaea* and *Christensenia* was tentative and they both showed long branches and possibly demonstrated convergent evolution in protein coding genes [1,10,63]. In our phylogenetic analysis, *Christensenia* was resolved as sister to the clade *Marattia* + *Angiopteris* s.l. with full support (ML-BS = 100; AS-PP = 1; QS = 1/−/1; ICA = 0.82–0.89), which was different from Lehtonen et al. [19]’s study that either recovered *Christensenia* as sister to *Marattia* (in plastid genome dataset) or as the second divergent clade sister to remaining genera except *Danaea* (in plastid genome and 77 phenotypic datasets). However, our inferred phylogeny based on multi-locus chloroplast genes also supports the same major clade position of Marattiaceae based on single-copy nuclear genes. In this case, two alternative explanations can be assumed: the phylogenetic reconstruction may be confounded by chloroplast RNA editing, and maternally inherited plastome representing only a single coalescent history may not reflect the species phylogenetic relationship [71,72,73,74,75]. The whole plastome is not “the holy grail” [76]; the effectiveness of plastome-scale data is ultimately reflected by the extent to which they reveal the “true” phylogenetic relationships of a given lineage [77]. Incomplete lineage sorting (ILS), introgressive hybridization, and horizontal gene transfer can also result in such high confliction [78]. Based on the phylogenetic analysis of transcriptome data and previous phylogenetic results, we determined that the most likely phylogenetic relationship of six genera in Marattiaceae is (*Danaea*, ((*Ptisana* + *Eupodium*), (*Christensenia*, (*Marattia* + *Angiopteris* s.l.)))).

### 3.2. Resolution of Angiopteris s.l.

In this study, a total of 10 samples representing 10 species of *Angiopteris* s.l. were included. Three well-supported clades (*Angiopteris* s.s., *Angiopteris sparsisora*, and the *Archangiopteris* group) were identified based on single-copy nuclear genes datasets and chloroplast genes (Figure 3a–d, Figure 7a,b, Figures S6a–d, S10–S12a,b and S16). Because of the similar morphology and poor phylogenetic resolution, *Angiopteris* s.s., *Archangiopteris*, *Macroglossum*, *Protomarattia* Hayata, and *Protangiopteris* Hayata were usually combined into *Angiopteris* s.l. [1,2,10,42,55,79]. In previous studies, *Angiopteris* s.s. often was not resolved as a monophyletic group [1,10,23,56,63]. *Macroglossum* has only one or two species with limited geographical distribution in Borneo and Sumatra [80]. *Archangiopteris* seems to be a monophyletic group [1] with about 10 species distributed in China and Vietnam [51,52,53]. We sampled four species and our phylogeny confirmed that the *Archangiopteris* group was a well-supported monophyletic clade in both nuclear gene datasets and chloroplast genes (Figure 3a–d, Figure 7a,b, Figures S6a–d, S10–S12a,b and S16). *Angiopteris* s.s. is paraphyletic, including two clades: the hybrid *An. sparsisora* and *Angiopteris* s.s., in all the nuclear gene datasets. *Angiopteris sparsisora* has been recognized as a putative hybrid between *Angiopteris* s.s. and the *Archangiopteris* group [42,57]. Ching and Wang [57] thought that *An. sparsisora* is more similar to *Angiopteris* s.s. morphologically and placed it in *Angiopteris*, a position that is also supported by our chloroplast genes (Figure S16). However, our phylogenetic analysis showed that *An. sparsisora* has a closer relationship with the *Archangiopteris* group instead of *Angiopteris* s.s. in all nuclear gene datasets. Morphologically, the *Archangiopteris* group is different from *Angiopteris* s.s. in having creeping rhizomes and often once-pinnate blades, the stipes with one or several (1–7) naked pulvini (presenting in whole growth phases of plants) [81].

*Archangiopteris* (=the *Archangiopteris* group) was established by Christ and Giesenhagen [62] based on *Ar. henryi* (=*An. latipinna*). The prefix “arch-” of *Archangiopteris* means primitive, representing *Archangiopteris* with more primitive morphological characters (or earlier differentiation) than *Angiopteris* s.s., which was also demonstrated in a chloroplast genes dataset with one outgroup (Figure S16). Such ideas were supported by some previous morphological studies and phylogenetic analysis [23,47,51,56,63]. However, our phylogenetic analysis recovered that *Angiopteris* s.s. was diverged earlier than the *Archangiopteris* group with full support value in all nuclear gene datasets (Figure 3a–d, Figure 7a,b, Figure 8, Figures S6a–d and S10–S12a,b). An estimated divergence time based on nuclear genes showed that the *Archangiopteris* group evolved ca. 27 Ma, nearly 10 Ma later than *Angiopteris*. It was also noted by some authors [51,64] who thought that the *Archangiopteris* group probably was derived from *Angiopteris* s.s. based on the taxonomy, geographical distribution, morphological, and anatomical features. In order to understand the phylogenetic relationship of *Angiopteris* s.l. well, potentially, some taxonomic ranks (nothosubgenus, subgenus, or section) in *Angiopteris* s.l. could be recognized and proposed, and hybridization events in Marattiaceae need to be further investigated in the future.

### 3.3. Phylogenetic Incongruence as Further Evidence for ILS and Hybridization

With different materials and methods, a large number of previous phylogenetic studies have yielded many incongruent topologies in Marattiaceae (Figure 1) [1,10,19,20,48,56,63]. In addition, our analyses showed that there was a very high level of heterogeneity among single-copy nuclear gene trees, and cytonuclear discordance with different phylogenetic relationships of Equisetaceae, Marattiaceae, Ophioglossiodeae, Polypodiidae, and Psilotaceae. While these inconsistencies have never been well explained, it is often believed that phylogenetic incongruence in plants is mainly due to hybridization and ILS [82,83,84,85,86].

Here, the phylogenetic relationship of Marattiaceae was inferred using coalescent methods, which also allow us to assess the ancient hybridization, introgression, and incomplete lineage sorting (ILS) [87]. The confliction within gene trees and among concatenation and coalescent trees at all topologies was detected in this study (Figure 2a,b, Figure 3a–d, Figure 5a,b, Figure 6a,b, Figure 7a,b, Figures S1a–d, S2a,b, S3–S5a,b, S6a–d and S7–S12a,b). At family level and genus level, nearly overlapping RF distances were detected between the empirical trees and the coalescent simulations, indicating that ILS alone could explain most of the gene tree discordance (Figure 5, Figure 6c,d, Figures S3–S5c,d and S7–S9c,d). Meanwhile, ILS is likely to apply regarding mainly reasons for family-level and genus-level conflict that diverged during rapid speciation events and/or had large population sizes [88]. We also detected inter-family and inter-genus geneflow among almost all lineages (Figure 3e, Figures S2, S3 and S5e). High hybridization frequency had been reported in ferns [89,90,91,92,93], and it is generally believed that hybridization and allopolyploidization are the main driving forces in the evolution of ferns [94]. Topology conflict in Marattiaceae at family level and genus level was potentially caused by ILS and hybridization together.

In *Angiopteris* s.l., our simulation analysis showed a large difference in both frequency and tree-to-tree distance between the coalescent simulations and the empirical trees (Figure 7c,d and Figures S10–S12c,d), and the ILS, therefore, cannot explain the unresolved phylogenetic relationship between *Angiopteris* s.s. and the *Archangiopteris* group. Potentially, gene flow is frequent between *Angiopteris* s.s. and the *Archangiopteris* group, leading to topological conflict. Hybridization analysis further suggested that *An. sparsisora* was of hybrid origin and was detected in all 20 examinations, with an inheritance probability of 0.371–0.506 from the *Angiopteris* s.s. lineage and 0.494–0.629 from the *Archangiopteris* group lineage (Figure 7e and Figures S10–S12e). Using the command CalGTProb, the greatest likelihood of the network was computed. It indicated that only one hybridization event was selected, which conflicts with the previous hypothesis of intergeneric hybridization event times (Table 3). *Angiopteris sparsisora* is endemic to a valley under evergreen broad-leaved forests in Xichou County, Fadou Town, which is located in southeastern Yunnan Province, China. Ching and Wang [57] have assumed that this distinct species is a natural hybrid between *Angiopteris* s.s. and the *Archangiopteris* group, with a number of intermediate morphological characteristics [42,57]. Ching and Wang [57] placed *An. sparsisora* in *Angiopteris* s.s. rather than in the *Archangiopteris* group because of the fact that, in overall impression, the new taxon appears more similar to the former than the latter. China and northern Vietnam was thought to be the original center, with high diversity of the *Archangiopteris* group. In this area, a large number of species of *Angiopteris* s.s. and the *Archangiopteris* group occur, growing side by side in great abundance. Another presumptive hybrid species, *An. bipinnata (=Ar. bipinnata),* was sampled in our study. We confirmed that *An. bipinnata* is a member of the *Archangiopteris* group. However, our analysis did not detect obvious gene flow from other lineages (Figure 7e and Figures S10–S12e). In *Angiopteris* s.s., some other hybrid species have also been reported. (e.g., *An. sugongii*, *An. itoi* (*=Ar. itoi*)), and it is necessary to investigate them with more materials and molecular data.

## 4. Materials and Methods

### 4.1. Taxon Sampling and RNA Sequencing

In this study, 26 samples representing 24 species (Table 1) were sampled, including almost all major lineages recognized in Marattiaceae plus outgroups. Eleven samples were newly generated in this study and 15 samples were obtained from GenBank SRA database (Table 1). In total, except *Eupodium* (a neotropical genus with three or four species [19,95]), all recognized genera of Marattiaceae in PPG I [2] were sampled. New transcriptome data were sequenced on an Illumina HiSeq2000 platform. Total RNA extraction, library preparation, raw data cleaning, and quality control were performed in 2018 at Majorbio, Shanghai, China.

### 4.2. Assembly and Single-Copy Orthologue Identification

Illumina adapter and poor-quality bases were removed from the raw reads of the transcriptomes using Fastp v0.12.4 [96] with default parameters. The cleaned data were used for de novo assembly using Trinity v2.8.5 [97] and only the longest transcripts were selected for subsequent analysis using Trinity’s own perl script to remove over-represented sequences. Minimap2 v2.17-r941 [98] and Samtools v1.11 [99] were chosen to filter the chloroplast and mitochondrial transcripts using the closest publicly available reference chloroplast genome and mitochondrion genome (Table S1) as references. CD-HIT-EST v4.8.1 [100] was used to further remove redundant contigs with a threshold of 0.99. TransDecoder v5.5.0 [101] was used to identify candidate coding regions. To cluster transcripts into orthologous genes, the OrthoFinder v2.3.8 [102,103] and SonicParanoid v1.3.8 [104] software were used to infer core-orthogroups based on all-against-all searches with default parameters and only single-copy orthologues present in all samples were selected for subsequent analyses. This resulted in 92 single-copy nuclear genes that shared with all 26 samples in total by using OrthoFinder and 187 single-copy nuclear genes that shared with all 26 samples in total by using SonicParanoid, respectively.

### 4.3. Nuclear Genes Dataset Generation and Phylogenetic Inference

Considering the later hybridization analysis with computational restrictions and by using different datasets to reconstruct the relationship of Marattiaceae, we first constructed 20 single-copy nuclear gene datasets composed of different species (Table S2) and discussed the phylogenetic relationship at the family level, genus level, species level, and the root of Marattiaceae. Each of the acquired amino acid sequences was aligned first then trimmed by using plugins Mafft v7.450 (set E-INS-i) [105,106] and trimAI v1.3 (set -automated1) [107] in PhyloSuite v1.2.2 [108], respectively. Given that recombination within loci might bias the inference of the species tree [109], we further used PhiPack v1.1 [110] to test the pairwise homoplasy index Φ for recombination with default sliding window size of 100 bp, and alignments that showed a strong signal of recombination with *p* ≤ 0.05 were removed from all subsequent phylogenetic analyses. Subsequently, for the concatenation approach in which all genes in a concatenated matrix are considered a single locus to infer phylogeny, sequences of aligned genes in each dataset were concatenated using the PhyloSuite plugins “Concatenate Sequence”. The concatenation ML tree inferred in IQ-tree v2.1.2 [111] with 10,000 ultrafast bootstraps [112] and performed model selection using the bias-corrected Akaike information criterion (AICc) in ModelFinder [113]. In addition, a coalescent approach was applied to estimate the species tree. Each gene tree was generated via IQ-tree with 1000 ultrafast bootstraps and the coalescent tree was inferred by Astral-II v5.7.8 [114]. In addition, TreeShrink v 1.3.7 [89] was selected to reduce the long branch attraction (LBA) influence. The retained sequences were used to construct the concatenation and coalescent trees with the same method mentioned above. Finally, the corresponding codon alignments were converted from amino acid sequences using PAL2NAL v14.0 [115] to generate datasets, remove recombination loci, reduce LBA, and for phylogeny reconstruction.

### 4.4. Organelle Genome Assembly, Locus Extraction, and Phylogenetic Analysis

Chloroplast and mitochondrion genes were obtained from assembly transcripts mapped to the closest available reference chloroplast and mitochondrion genome (Table S1) in Geneious Prime [116] with the parameter settings in the “Geneious RNA” program as highest sensitivity. Annotation was subsequently conducted in Geneious [set 90% similarity]. Then, we filled in the gaps with ‘N’ for all of the protein-coding genes and extracted. Each of the acquired protein-coding genes was aligned first then trimmed by using plugins Mafft (set E-INS-i) and trimAI (set -automated1) in PhyloSuite, respectively. For the concatenated matrix, we constructed chloroplast genes dataset (CT), mitochondrial genes dataset (MT), and chloroplast genes and mitochondrial genes combined dataset (CM) and then used the 5000 ultrafast bootstrap replicates for branch support in IQ-tree.

### 4.5. Concordance, ILS Simulation, and Hybridization Inference

We used two approaches to explore discordance among nuclear gene trees. First, we mapped the individual gene trees onto the coalescent tree and calculated the internode certainty all (ICA [117]) value to quantify the degree of conflict on each node and the number of conflicting and concordant bipartition on each node of the coalescent trees using the gene trees with maximum likelihood bootstrap supports (MLBS) values at least 50% for the corresponding node. Both ICA and conflicting/concordant bipartitions were calculated with Phyparts [118]. Then, we used quartet sampling (QS [119]) to distinguish strong conflict from weakly supported branches using the concatenation tree with 1000 replicates. For the ILS analysis, we used Dendropy v4.5.2 [120] to simulate gene trees using the species tree as a guide tree and generated a total of 20,000 gene trees. Then, we calculated the Robinson-Foulds (RF) distance between the species tree and each simulated or observed gene tree by using the workflow of Wang et al. [82]. To test the hypothesis of hybridization, we used PhyloNet v3.8.0 [121] to infer an evolutionary network with the command “InferNetworks_ML” [122] from individual gene trees. Due to computational restrictions, a maximum of five reticulation events was set and run with 50 runs to ensure accuracy. To estimate the optimal number of hybridizations, we computed the likelihood scores of the given individual gene trees using the command “CalGTProb” [123]. The networks were visualized in Dendroscope v3.8.1 [124].

### 4.6. Age Estimation

Divergence time estimations were performed based on the concatenated 16-O-cds dataset using penalized likelihood in treePL v2.6.3 [125]. We were mainly concerned with the chronological order of divergence of the two lineages of *Angiopteris* s.s. and the *Archangiopteris* group. We used two calibration points based on the estimation of Lehtonen et al. [19], who applied a parsimony-based total-evidence dating approach, which suggested a Triassic age for the extant crown group, which is consistent with the fossil records that have been found so far [12,49,126,127]: (1) an age estimate ranging from 307 Ma to 320 Ma for the Marattiales, and (2) the split between *Danaea* and the most recent common ancestor of the rest extant species of Marattiaceae was estimated in the Late Triassic (201–236 Ma). Following the guidelines of Maurin [128], cross-validation analysis was conducted with rate-smoothing values from 1010 to 10–30 and a “cvmultstep” of 0.1 and resulted in an optimal smoothing parameter of 0.00001. Lastly, used TreeAnnotater v2.4.7 to summarize all dated 1000 bootstrap replicates into a maximum clade credibility (MCC) tree with 0% of burn-in and mean node heights. The MCC tree was displayed in FigTree v1.4.3 [129].

## 5. Conclusions

Using different phylogenetic analyses, the phylogeny of Marattiaceae was reconstructed with a dataset from 26 transcriptomes. In general, our results are consistent with previous phylogenetic results [1,10]. The most likely phylogenetic relationship of six genera in Marattiaceae is (*Danaea*, ((*Ptisana* + *Eupodium*), (*Christensenia*, (*Marattia* + *Angiopteris* s.l.)))). Moreover, some new insights have been revealed for hybridization in *Angiopteris* s.l. The *Archangiopteris* group (=*Archangiopteris*) forms a well-supported clade that is nested within *Angiopteris* s.s. The putative hybrid species *Angiopteris sparsisora* between *Angiopteris* s.l. and the *Archangiopteris* group was confirmed by the network analysis and maternal chloroplast genes. ILS and gene flow could be the main reason for the puzzled systematists at family level, genus level, and species level in Marattiaceae.

## Figures and Tables

**Table 1:** Voucher information of taxa included in this study.

Family	Genera (PPG I)	Species	Voucher Data	Locations or Sample Provider	Citation
Marattiaceae	*Angiopteris*	*An. caudatiformis* Hieronymus	AN_CA (PYU)	Yunnan, China,	This study
Marattiaceae	*Angiopteris*	*An. fokiensis* Hieronymus	AN_FO (PYU)	Yunnan, China	This study
Marattiaceae	*Angiopteris*	*An. hokouensis* Ching	AN_HO (PYU)	Yunnan, China	This study
Marattiaceae	*Angiopteris*	*An. sparsisora* Ching	AN_SP (PYU)	Yunnan, China	This study
Marattiaceae	*Angiopteris*	*An. wangii* Ching	AN_WA (PYU)	Yunnan, China	This study
Marattiaceae	*Angiopteris*	*An. yunnanensis* Hieronymus	AN_YN (PYU)	Yunnan, China	This study
Marattiaceae	*Angiopteris*	* *An. bipinnata* (Ching) J.M. Camus (=*Ar. bipinnata* Ching)	AN_BP (PYU)	Yunnan, China	This study
Marattiaceae	*Angiopteris*	* *An. latipinna* Z.R. He, W.M. Chu & Christenhusz (=*Ar. henryi* Christ & Giesenhagen)	AN_LA (PYU)	Yunnan, China	This study
Marattiaceae	*Angiopteris*	* *An. chingii* J. M. Camus (=*Ar. hokouensis* Ching)	AN_CH (PYU)	Yunnan, China	This study
Marattiaceae	*Angiopteris*	* *An. subrotundata* (Ching) Z.R. He & Christenhusz (=*Ar. subrotundata* Ching)	AN_SU (PYU)	Yunnan, China	This study
Marattiaceae	*Christensenia*	*Christensenia aesculifolia* (Blume) Maxon	CH_AE (PYU)	Yunnan, China	This study
Marattiaceae	*Christensenia*	*Christensenia aesculifolia* (Blume) Maxon	K024247 (KBCC)	Dr. Ceceilia Koo of Botanic Conservation Center	[34]
Marattiaceae	*Danaea*	*Danaea nodosa* (L.) Sm.	Vasco 879 (NY)	Sample Provider: Alejandra Vasco	1 kp., 2019
Marattiaceae	*Danaea*	*Danaea nodosa* (L.) Sm.	K034856(KBCC)	Dr. Ceceilia Koo of Botanic Conservation Center	[34]
Marattiaceae	*Marattia*	*Marattia* sp.	NYBG1295/78-A (NYBG)	Sample Provider: D. W. Stevenson	1 kp., 2019
Marattiaceae	*Ptisana*	*Ptisana pellucida* (C.Presl) Murdock	K013208(FUS)	Dr. Ceceilia Koo of Botanic Conservation Center	[34]
Equisetaceae	*Equisetum*	*Equisetum arvense* L.	xp705 (FUS)	Zhejiang, China	[34]
Equisetaceae	*Equisetum*	*Equisetum diffusum* D.Don	RS-107 (CSH)	Sichuan, China	[39]
Psilotaceae	*Psilotum*	*Psilotum nudum* (L.) P.Beauv.	xp703 (FUS)	Guangxi, China	[34]
Ophioglossaceae	*Ophioglossum*	*Ophioglossum thermale* Kom.	xp746 (FUS)	Shanghai, China	[34]
Ophioglossaceae	*Ophioglossum*	*Ophioglossum vulgatum* L.	RS-84 (CSH)	Guangdong, China	[39]
Osmundaceae	*Osmundastrum*	*Osmundastrum cinnamomeum* (L.) C.Presl	Unknown (NYBG)	Sample Provider: D. W. Stevenson	1 kp., 2019
Osmundaceae	*Osmunda*	*Osmunda japonica* Thunb.	RS38 (CSH)	Shanghai, China	[39]
Hymenophyllaceae	*Hymenophyllum*	*Hymenophyllum holochilum* (Bosch) C.Chr.	Kuo4290 (TAFI)	Taiwan, China	[34]
Hymenophyllaceae	*Vandenboschia*	*Vandenboschia speciosa* (Willd.) G.Kunkel	Unknown	Alcornocales Natural Park (Cádiz, Spain): Valdeinfierno (VDI).	[69]
Amborellaceae	*Amborella*	*Amborella trichopoda* Baill.	Unknown	Unknown	Unknown

Note: An. *Angiopteris*; Ar. *Archangiopteris*; * show these species have been recognized as the members of *Archangiopteris* historically.

**Table 2:** Datasets’ statistics.

Dataset	26-O-aa	26-O-cds	26-S-aa	26-S-cds	16-O-aa	16-O-cds	16-S-aa	16-S-cds	Family-O-aa	Family-O-cds
Number of Taxon	26	26	26	26	16	16	16	16	11	11
Number of Gene	65	80	147	162	474	595	782	947	749	834
Number of Sites	23,136	78,828	62,321	194,713	212,344	679,941	381,413	1,189,191	351,399	1,093,200
Number of Informative sites	11,278	43,172	29,032	103,057	32,577	83,068	55,719	137,762	148,631	523,878
Number of Invariable sites	8888	26,923	24,912	69,474	157,262	541,874	285,182	955,281	144,968	416,330
Sequence Length	23,136	78,828	62,321	194,713	212,344	679,941	381,413	1,189,191	351,399	1,093,200
Pairwise Identity (%)	71.10	70.70	69.50	69.20	81.30	83.70	78.90	81.30	62.40	62.50
Identical Sites (%)	14.40	12.60	12.10	10.70	27.10	32.00	25.40	30.20	27.90	24.60
Average GC content (%)	−	46.60	−	46.40	−	47.30	−	46.90	−	45.50
Max Sequence Length (bp)	22,918	77,973	60,898	190,133	207,882	665,631	367,727	1,147,431	344,823	1,072,395
Min Sequence Length (bp)	11,416	38,560	25,970	84,138	93,695	323,922	159,932	542,583	296,857	917,580
Mean coverage (%)	25.98	26.00	25.99	25.99	16.00	16.00	16.00	16.00	11.00	11.00
Dataset	Family-S-aa	Family-S-cds	Genus-O-aa	Genus-O-cds	Genus-S-aa	Genus-S-cds	Species-O-aa	Species-O-cds	Species-S-aa	Species-S-cds
Number of Taxon	11	11	9	9	9	9	11	11	11	11
Number of Gene	1293	1397	700	911	1105	1389	541	1058	616	1216
Number of Sites	678,528	2,102,052	314,325	1,032,691	532,963	1,702,007	244,990	967,773	269,150	1,094,673
Number of Informative sites	289,393	1,012,805	45,580	118,504	73,153	186,245	4850	10,908	5510	12,700
Number of Invariable sites	273,670	785,106	235,281	830,260	401,729	1,375,289	232,965	939,305	254,861	1,060,028
Sequence Length	678,528	2,102,052	314,325	1,032,691	532,963	1,702,007	244,990	967,773	269,150	1,094,673
Pairwise Identity (%)	60.70	61.00	71.80	75.50	69.90	73.60	84.90	89.10	84.60	89.20
Identical Sites (%)	25.90	22.80	27.30	33.10	26.50	31.70	38.80	54.50	42.80	58.00
Average GC content (%)	−	45.30	−	46.90	−	46.60	−	47.50	−	47.50
Max Sequence Length (bp)	661,831	2,050,271	305,614	1,005,724	508,729	1,631,249	239,958	952,194	261,892	1,073,154
Min Sequence Length (bp)	575,170	1,774,443	133,950	488,227	221,802	779,205	111,063	578,586	136,543	702,030
Mean coverage (%)	11.00	11.00	9.00	9.00	9.00	9.00	11.00	11.00	11.00	11.00

Note: “O” and “S” mean performed by Orothofinder and SonicParanoid. “aa” and “cds” mean amino acid sequence and nucleotide sequence.

**Table 3:** Likelihood scores of networks corresponding to the best five network searches. The bold values show the best network.

Total Log Probability	Family-O-aa	Family-O-cds	Family-S-aa	Family-S-cds	Genus-O-aa	Genus-O-cds
Network 1	−6190.5703	−3501.058081	−11295.45488	−5950.690602	−744.3201955	−457.4195646
Network 2	−6113.875518	−3227.002002	−11199.63915	−5872.642211	−743.1046667	−487.2788227
Network 3	−6231.19481	−3287.813375	−11385.39823	−5950.690602	−720.0360015	−487.2788227
Network 4	**−5643.849581**	**−3323.11141**	**−10901.25444**	**−5158.20478**	−743.3484675	**−452.0974283**
Network 5	−6165.723153	−3484.299045	−11033.73158	−5699.184902	**−712.7726379**	−510.6857333
Total log probability	Genus-S-aa	Genus-S-cds	Species-O-aa	Species-O-cds	Species-S-aa	Species-S-cds
Network 1	**−1357.70562**	−772.4172772	**−247.1150431**	**−199.7265792**	**−328.9024602**	−267.86003727524957
Network 2	−1358.256057	**−772.0158611**	−249.0607795	−213.7213554	−332.0709943	**−267.86003727524945**
Network 3	−1358.256057	−773.8058876	−247.117924	−220.5996779	−331.5036725	−272.798912
Network 4	−1377.690453	−808.015031	−249.0611516	−199.8006603	−331.5036725	−268.2104459
Network 5	−1389.124744	−808.015031	−249.0611364	−226.4899819	−328.9071446	−300.2662086

Note: the bold values indicated the best network using the command “CalGTProb” in PhyloNet.

## Data Availability

The multiple sequence alignments, concatenated alignments, and phylogenetic trees for this study are publicly available at Figshare repository: https://doi.org/10.6084/m9.figshare.21707546.

## Supplementary Materials

The following supporting information can be downloaded at: https://www.mdpi.com/article/10.3390/plants12122237/s1. Table S1: The references of complete organelle genome used in this study. Table S2: Sample composition of different datasets. Table S3: Information of transcriptomes included in this study. Figure S1: Phylogenomic analysis of concatenation-based trees from the 26-O-aa and 26-O-cds datasets in Marattiaceae (a,b. 26-O-aa datasets, c,d. 26-O-cds datasets); a,c. astral-II posterior probabilities (AS-PP) and internode certainty all (ICA) values are shown above branches, and numbers of gene trees (concordant/conflicting) were shown below branches. Pie charts present the proportion of congruent gene trees that supports that clade (blue), the proportion of discordant gene trees of the main alternative topology for that clade (green), the proportion of discordant trees for the remaining alternative topologies (red), the proportion of uninformative gene trees (dark gray; bootstrap support <50%), and the proportion of missing data (light gray); b,d. maximum likelihood bootstrap support (ML-BS) is shown above the branches, and quartet sampling internal node scores (quartet concordance/quartet differential/quartet informativeness) are shown below branches. Figure S2: Phylogenetic analysis of species trees based on 26-S-cds in Marattiaceae. a. Astral-II posterior probabilities (AS-PP) and internode certainty all (ICA) values are shown above branches, and numbers of gene trees (concordant/conflicting) were shown below branches. Pie charts present the proportion of congruent gene trees that supports that clade (blue), the proportion of discordant gene trees of the main alternative topology for that clade (green), the proportion of discordant trees for the remaining alternative topologies (red), the proportion of uninformative gene trees (dark gray; bootstrap support <50%), and the proportion of missing data (light gray). b. Maximum likelihood bootstrap support (ML-BS) is shown above the branches, and quartet sampling internal node scores (quartet concordance/quartet differential/quartet informativeness) are shown below branches. Figure S3: a,b. Phylogenetic analysis of species tree with Family-O-cds dataset in Marattiaceae (a. Astral-II posterior probabilities (AS-PP) and internode certainty all (ICA) scores are shown above branches and numbers of gene trees (concordant/conflicting) are shown next to the nodes; b. maximum likelihood bootstrap support (ML-BS) is shown above the branches. Quartet sampling internal node scores (quartet concordance/quartet differential/quartet informativeness) are shown below branches); c. distributions of topology frequencies of observed and simulated gene trees based on Family-O-cds dataset; d. distribution of Robinson-Foulds distances of the simulated and observed gene trees to the coalescent tree; e. species network inferred from PhyloNet pseudolikelihood analyses with one to five hybridization events based on Family-O-cds dataset. Red and blue curved branches indicate the minor and major edges of hybrid nodes, respectively. Numbers next to curved branches indicate inheritance probabilities for each hybrid node. Figure S4: a,b. Phylogenetic analysis of species tree with Family-S-aa dataset in Marattiaceae (a. Astral-II posterior probabilities (AS-PP) and internode certainty all (ICA) scores are shown above branches and numbers of gene trees (concordant/conflicting) are shown next to the nodes; b. maximum likelihood bootstrap support (ML-BS) is shown above the branches. Quartet sampling internal node scores (quartet concordance/quartet differential/quartet informativeness) are shown below branches); c. distributions of topology frequencies of observed and simulated gene trees based on Family-S-aa dataset; d. distribution of Robinson-Foulds distances of the simulated and observed gene trees to the coalescent tree; e. species network inferred from PhyloNet pseudolikelihood analyses with one to five hybridization events based on Family-S-aa dataset. Red and blue curved branches indicate the minor and major edges of hybrid nodes, respectively. Numbers next to curved branches indicate inheritance probabilities for each hybrid node. Figure S5: a,b. Phylogenetic analysis of species tree with Family-S-cds dataset in Marattiaceae (a. Astral-II posterior probabilities (AS-PP) and internode certainty all (ICA) scores are shown above branches and numbers of gene trees (concordant/conflicting) are shown next to the nodes; b. maximum likelihood bootstrap support (ML-BS) is shown above the branches. Quartet sampling internal node scores (quartet concordance/quartet differential/quartet informativeness) are shown below branches); c. distributions of topology frequencies of observed and simulated gene trees based on Family-S-cds dataset; d. distribution of Robinson-Foulds distances of the simulated and observed gene trees to the coalescent tree; e. species network inferred from PhyloNet pseudolikelihood analyses with one to five hybridization events based on Family-S-cds dataset. Red and blue curved branches indicate the minor and major edges of hybrid nodes, respectively. Numbers next to curved branches indicate inheritance probabilities for each hybrid node. Figure S6: Phylogenomic analysis of species trees based on 16-O-aa and 16-S-aa datasets in Marattiaceae (a,b. 16-O-aa datasets; c,d. 16-S-aa datasets). a,c. Astral-II posterior probabilities (AS-PP) and internode certainty all (ICA) values are shown above branches, and numbers of gene trees (concordant/conflicting) were shown below branches. Pie charts present the proportion of congruent gene trees that supports that clade (blue), the proportion of discordant gene trees of the main alternative topology for that clade (green), the proportion of discordant trees for the remaining alternative topologies (red), the proportion of uninformative gene trees (dark gray; bootstrap support <50%), and the proportion of missing data (light gray); b,d. maximum likelihood bootstrap support (ML-BS) is shown above the branches, and quartet sampling internal node scores (quartet concordance/quartet differential/quartet informativeness) are shown below branches. Figure S7: a,b. Phylogenetic analysis of species tree with Genus-O-aa dataset in Marattiaceae (a. Astral-II posterior probabilities (AS-PP) and internode certainty all (ICA) scores are shown above branches and numbers of gene trees (concordant/conflicting) are shown next to the nodes; b. maximum likelihood bootstrap support (ML-BS) is shown above the branches. Quartet sampling internal node scores (quartet concordance/quartet differential/quartet informativeness) are shown below branches); c. distributions of topology frequencies of observed and simulated gene trees based on Genus-O-aa dataset; d. distribution of Robinson-Foulds distances of the simulated and observed gene trees to the coalescent tree; e. species network inferred from PhyloNet pseudolikelihood analyses with one to five hybridization events based on Genus-O-aa dataset. Red and blue curved branches indicate the minor and major edges of hybrid nodes, respectively. Numbers next to curved branches indicate inheritance probabilities for each hybrid node. Figure S8: a,b. Phylogenetic analysis of species tree with Genus-S-aa dataset in Marattiaceae (a. Astral-II posterior probabilities (AS-PP) and internode certainty all (ICA) scores are shown above branches and numbers of gene trees (concordant/conflicting) are shown next to the nodes; b. maximum likelihood bootstrap support (ML-BS) is shown above the branches. Quartet sampling internal node scores (quartet concordance/quartet differential/quartet informativeness) are shown below branches); c. distributions of topology frequencies of observed and simulated gene trees based on Genus-S-aa dataset; d. distribution of Robinson-Foulds distances of the simulated and observed gene trees to the coalescent tree; e. species network inferred from PhyloNet pseudolikelihood analyses with one to five hybridization events based on Genus-S-aa dataset. Red and blue curved branches indicate the minor and major edges of hybrid nodes, respectively. Numbers next to curved branches indicate inheritance probabilities for each hybrid node. Figure S9: a,b. Phylogenetic analysis of species tree with Genus-S-cds dataset in Marattiaceae (a. Astral-II posterior probabilities (AS-PP) and internode certainty all (ICA) scores are shown above branches and numbers of gene trees (concordant/conflicting) are shown next to the nodes; b. maximum likelihood bootstrap support (ML-BS) is shown above the branches. Quartet sampling internal node scores (quartet concordance/quartet differential/quartet informativeness) are shown below branches); c. distributions of topology frequencies of observed and simulated gene trees based on Genus-S-cds dataset; d. distribution of Robinson-Foulds distances of the simulated and observed gene trees to the coalescent tree; e. species network inferred from PhyloNet pseudolikelihood analyses with one to five hybridization events based on Genus-S-cds dataset. Red and blue curved branches indicate the minor and major edges of hybrid nodes, respectively. Numbers next to curved branches indicate inheritance probabilities for each hybrid node. Figure S10: a,b. Phylogenetic analysis of species tree with Species-O-cds dataset in Marattiaceae (a. Astral-II posterior probabilities (AS-PP) and internode certainty all (ICA) scores are shown above branches and numbers of gene trees (concordant/conflicting) are shown next to the nodes; b. maximum likelihood bootstrap support (ML-BS) is shown above the branches. Quartet sampling internal node scores (quartet concordance/quartet differential/quartet informativeness) are shown below branches); c. distributions of topology frequencies of observed and simulated gene trees based on Species-O-cds dataset; d. distribution of Robinson-Foulds distances of the simulated and observed gene trees to the coalescent tree; e. species network inferred from PhyloNet pseudolikelihood analyses with one to five hybridization events based on Species-O-cds dataset. Red and blue curved branches indicate the minor and major edges of hybrid nodes, respectively. Numbers next to curved branches indicate inheritance probabilities for each hybrid node. Figure S11: a,b. Phylogenetic analysis of species tree with Species-S-aa dataset in Marattiaceae (a. Astral-II posterior probabilities (AS-PP) and internode certainty all (ICA) scores are shown above branches and numbers of gene trees (concordant/conflicting) are shown next to the nodes; b. maximum likelihood bootstrap support (ML-BS) is shown above the branches. Quartet sampling internal node scores (quartet concordance/quartet differential/quartet informativeness) are shown below branches); c. distributions of topology frequencies of observed and simulated gene trees based on Species-S-aa dataset; d. distribution of Robinson-Foulds distances of the simulated and observed gene trees to the coalescent tree; e. species network inferred from PhyloNet pseudolikelihood analyses with one to five hybridization events based on Species-S-aa dataset. Red and blue curved branches indicate the minor and major edges of hybrid nodes, respectively. Numbers next to curved branches indicate inheritance probabilities for each hybrid node. Figure S12: a,b. Phylogenetic analysis of species tree with Species-S-cds dataset in Marattiaceae (a. Astral-II posterior probabilities (AS-PP) and internode certainty all (ICA) scores are shown above branches and numbers of gene trees (concordant/conflicting) are shown next to the nodes; b. maximum likelihood bootstrap support (ML-BS) is shown above the branches. Quartet sampling internal node scores (quartet concordance/quartet differential/quartet informativeness) are shown below branches); c. distributions of topology frequencies of observed and simulated gene trees based on Species-S-cds dataset; d. distribution of Robinson-Foulds distances of the simulated and observed gene trees to the coalescent tree; e. species network inferred from PhyloNet pseudolikelihood analyses with one to five hybridization events based on Species-S-cds dataset. Red and blue curved branches indicate the minor and major edges of hybrid nodes, respectively. Numbers next to curved branches indicate inheritance probabilities for each hybrid node. Figure S13: Maximum likelihood phylogram derived from CT dataset. Bootstrap support values (ML-BS) are shown above the branches. Figure S14: Maximum likelihood phylogram derived from MT dataset. Bootstrap support values (ML-BS) are shown above the branches. Figure S15: Maximum likelihood phylogram derived from CM dataset. Bootstrap support values (ML-BS) are shown above the branches. Figure S16: Relationship of *Angiopteris* s.s. with one outgroup based on chloroplast genes. Bootstrap support values (ML-BS) are shown above the branches.
