# Supplementary material for: Transcriptome-Based Study on the Phylogeny and Hybridization of Marattialean Ferns (Marattiaceae)
Source: Plants (Basel). 2023 Jun 7;12(12):2237. doi: 10.3390/plants12122237 (PMC10301251; doi:10.3390/plants12122237)
Supplement: Supplementary file 1 [file plants-12-02237-s001.zip › TableS1-S3.pdf]

Table S1. The references of complete organelle genome used in this study.

Species	Genbank number	Organelle	Study
Psilotum nudum	KX171638	mitochondrion	Guo *et al*., 2017
Psilotum nudum	KX171639	mitochondrion	Guo *et al*., 2017
Ophioglossum californicum	NC030900	mitochondrion	Guo *et al*., 2017
Angiopteris evecta	DQ821119	chloroplast	Roper *et al*., 2007
Christensenia aesculifolia	NC044756	chloroplast	Liu *et al*., 2019
Danaea sellowiana	NC051976	chloroplast	Lehtonen *et al*., 2020
Equisetum arvense	NC014699	chloroplast	Karol *et al*., 2010
Hymenophyllum holochilum	NC039753	chloroplast	Kuo *et al*., 2018
Marattia laxa	NC051979	chloroplast	Lehtonen *et al*., 2020
Ophioglossum californicum	NC020147	chloroplast	Grewe *et al*., 2013
Osmunda mildei	MZ292715	chloroplast	Zuo *et al*., 2021
Osmundastrum cinnamomeum	NC024157	chloroplast	Kim *et al*., 2014
Psilotum nudum	KC117179	chloroplast	Grewe *et al*., 2013
Ptisana novoguineensis	NC051980	chloroplast	Lehtonen *et al*., 2020
Vandenboschia speciosa	NC041000	chloroplast	Ruiz-Ruano *et al*., 2018

Table S2. Sample composition of different datasets.

Species	SRA number	26-O-aa	26-O-cds	26-S-aa	26-S-cds	16-O-aa	16-O-cds	16-S-aa	16-S-cds	Family-O-aa	Family-O-cds
Angiopteris caudatiformis√	This study	√	√	√	√	√	√	√	√		
Angiopteris fokiensis	This study	√	√	√	√	√	√	√	√		
Angiopteris hokouensis	This study	√	√	√	√	√	√	√	√		
Angiopteris sparsisora	This study	√	√	√	√	√	√	√	√		
Angiopteris wangii	This study	√	√	√	√	√	√	√	√		
Angiopteris yunnanensis	This study	√	√	√	√	√	√	√	√		
Angiopteris bipinnate	This study	√	√	√	√	√	√	√	√		
Angiopteris latipinna	This study	√	√	√	√	√	√	√	√		
Angiopteris chingii	This study	√	√	√	√	√	√	√	√		
Angiopteris subrotundata	This study	√	√	√	√	√	√	√	√		
Christensenia aesculifolia	This study	√	√	√	√	√	√	√	√	√	√
Christensenia aesculifolia	SRR6920618	√	√	√	√	√	√	√	√		
Danaea nodosa	ERR2041196	√	√	√	√	√	√	√	√		
Danaea nodosa	SRR6920621	√	√	√	√	√	√	√	√	√	√
Marattia sp.	ERR3487391	√	√	√	√	√	√	√	√		
Ptisana pellucida	SRR6920616	√	√	√	√	√	√	√	√		
Equisetum arvense	SRR6920624	√	√	√	√					√	√
Equisetum diffusum	SRR2103706	√	√	√	√					√	√
Psilotum nudum	SRR6920623	√	√	√	√					√	√
Ophioglossum thermale	SRR6920622	√	√	√	√					√	√
Ophioglossum vulgatum	SRR2103720	√	√	√	√					√	√
Osmundastrum cinnamomeum	ERR2041191	√	√	√	√					√	√
Osmunda japonica	SRR2103721	√	√	√	√					√	√

Hymenophyllum holochilum	SRR6920702	√	√	√	√					√	√
Vandenboschia speciosa	ERR2020652	√	√	√	√						
Amborella trichopoda	SRR15206987	√	√	√	√					√	√

Species	SRA number	Family-S-aa	Family-S-cds	Genus-O-aa	Genus-O-cds	Genus-S-aa	Genus-S-cds	Species-O-aa	Species-O-cds	Species-S-aa	Species-S-cds
Angiopteris caudatiformis√	This study							√	√	√	√
Angiopteris fokiensis	This study			√	√	√	√	√	√	√	√
Angiopteris hokouensis	This study							√	√	√	√
Angiopteris sparsisora	This study			√	√	√	√	√	√	√	√
Angiopteris wangii	This study							√	√	√	√
Angiopteris yunnanensis	This study							√	√	√	√
Angiopteris bipinnate	This study							√	√	√	√
Angiopteris latipinna	This study							√	√	√	√
Angiopteris chingii	This study							√	√	√	√
Angiopteris subrotundata	This study			√	√	√	√	√	√	√	√
Christensenia aesculifolia	This study	√	√	√	√	√	√				
Christensenia aesculifolia	SRR6920618			√	√	√	√				
Danaea nodosa	ERR2041196			√	√	√	√				
Danaea nodosa	SRR6920621	√	√	√	√	√	√				
Marattia sp.	ERR3487391			√	√	√	√	√	√	√	√
Ptisana pellucida	SRR6920616										
Equisetum arvense	SRR6920624	√	√								
Equisetum diffusum	SRR2103706	√	√								
Psilotum nudum	SRR6920623	√	√								
Ophioglossum thermale	SRR6920622	√	√								
Ophioglossum vulgatum	SRR2103720	√	√								

Osmundastrum cinnamomeum	ERR2041191	√	√
Osmunda japonica	SRR2103721	√	√
Hymenophyllum holochilum	SRR6920702	√	√
Vandenboschia speciosa	ERR2020652		
Amborella trichopoda	SRR15206987	√	√

Note. “O” and “S” means performed by Orothofinder and SonicParanoid. “aa” and “cds” means amino acid sequence and nucleotide sequence.

Table S3. Information of transcriptomes included in this study

Species	Raw reads	Raw bases	Percent GC of raw data	Filtered reads	Filtered bases	Percent GC of filtered data
Angiopteris caudatiformis	50,727,232	7,659,812,032	46.40%	47,027,154	7,071,635,285	46.78%
Angiopteris fokiensis	49,708,970	7,506,054,470	46.85%	46,061,868	6,916,894,226	47.21%
Angiopteris hokouensis	49,909,892	7,536,393,692	46.38%	46,182,844	6,939,795,547	46.76%
Angiopteris sparsisora	52,742,160	7,964,066,160	46.60%	48,767,488	7,325,393,780	46.96%
Angiopteris wangii	51,232,230	7,736,066,730	47.02%	47,540,386	7,144,092,364	47.42%
Angiopteris yunnanensis	47,412,096	7,159,226,496	47.07%	43,937,712	6,599,561,095	47.47%
**Angiopteris bipinnate*	47,475,778	7,168,842,478	46.84%	44,062,764	6,620,698,777	47.24%
**Angiopteris latipinna*	46,204,608	6,976,895,808	46.78%	42,900,906	6,443,400,757	47.17%
**Angiopteris chingii*	45,021,794	6,798,290,894	46.59%	41,613,264	6,256,095,110	46.92%
**Angiopteris subrotundata*	56,347,342	8,508,448,642	46.82%	52,013,624	7,796,020,285	47.08%
Christensenia aesculifolia	48,211,488	7,279,934,688	47.30%	44,635,604	6,705,581,279	47.70%
Christensenia aesculifolia	23,400,326	2,363,432,926	46.29%	21,892,288	2,209,225,642	46.04%
Danaea nodosa	31,192,790	2,807,351,100	48.95%	31,192,352	2,805,994,132	48.95%
Danaea nodosa	48,290,300	7,243,545,000	47.62%	46,770,954	6,278,417,128	47.19%
Marattia sp.	22,432,808	2,018,952,720	44.87%	22,432,080	2,017,698,528	44.87%
Ptisana pellucida	31,785,910	3,210,376,910	47.68%	29,564,784	2,982,169,052	47.37%
Equisetum arvense	19,888,142	2,008,702,342	46.83%	18,801,736	1,895,566,522	46.75%
Equisetum diffusum	37,143,940	4,680,136,440	48.28%	33,109,634	4,078,250,170	48.26%
Psilotum nudum	17,437,634	1,761,201,034	45.94%	16,595,954	1,673,419,188	45.88%
Ophioglossum thermale	30,567,136	3,087,280,736	45.23%	28,574,566	2,881,902,694	45.17%
Ophioglossum vulgatum	36,397,236	4,586,051,736	51.29%	33,873,546	4,200,456,856	51.43%

Osmundastrum cinnamomeum	19,656,280	1,769,065,200	48.23%	19,656,160	1,767,620,120	48.23%
Osmunda japonica	34,138,716	4,301,478,216	48.68%	31,748,310	3,937,276,206	48.71%
Hymenophyllum holochilum	20,644,362	3,096,654,300	48.49%	20,077,500	2,825,831,220	48.15%
Vandenboschia speciosa	66,346,550	6,701,001,550	47.46%	62,756,378	6,319,062,682	47.30%
Amborella trichopoda	79,878,588	7,941,924,719	44.68%	78,938,126	7,848,172,731	44.69%

*Show these species have been recognized as the members of *Archangiopteris* historically.
