# Supplementary figures and images for: Transcriptome-Based Study on the Phylogeny and Hybridization of Marattialean Ferns (Marattiaceae)
Source: Plants (Basel). 2023 Jun 7;12(12):2237. doi: 10.3390/plants12122237 (PMC10301251; doi:10.3390/plants12122237)

(a)

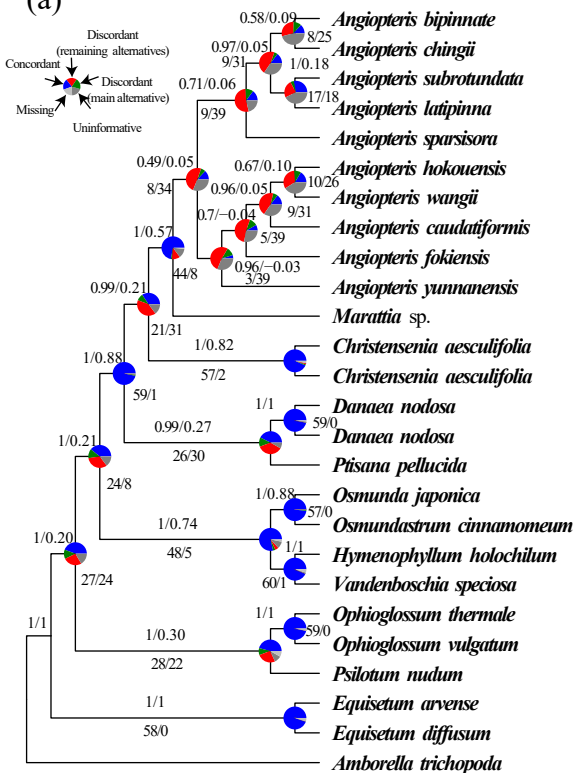

(b)

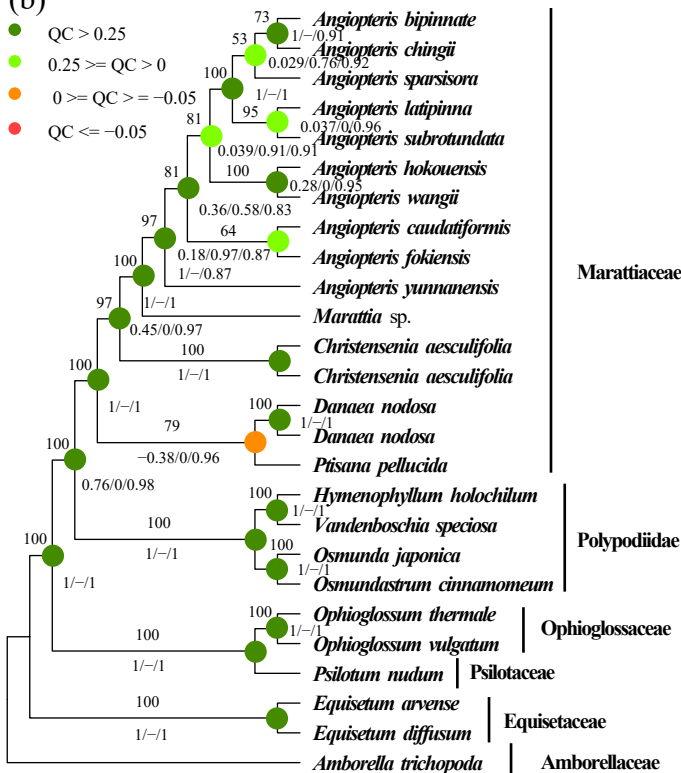

(c)

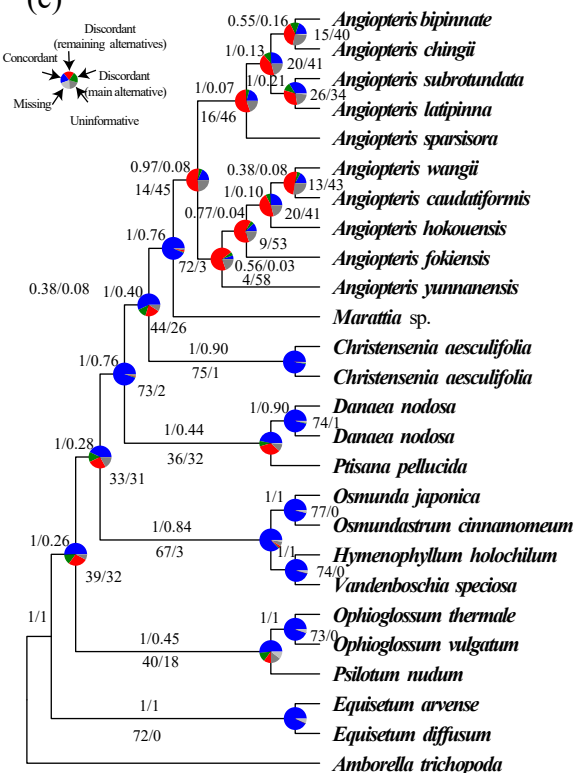

(c)

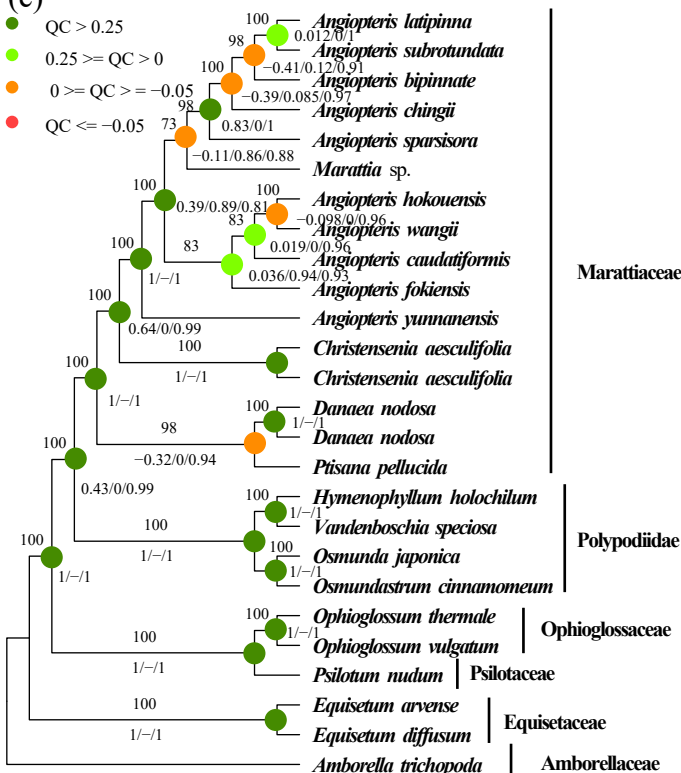

Supplement: Supplementary file 1 [file plants-12-02237-s001.zip › FigS1.pdf]

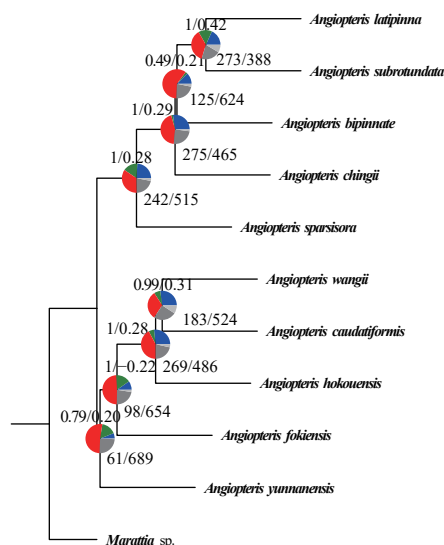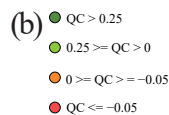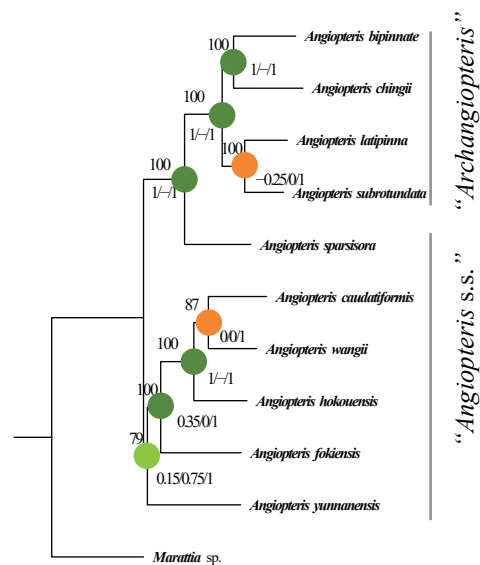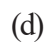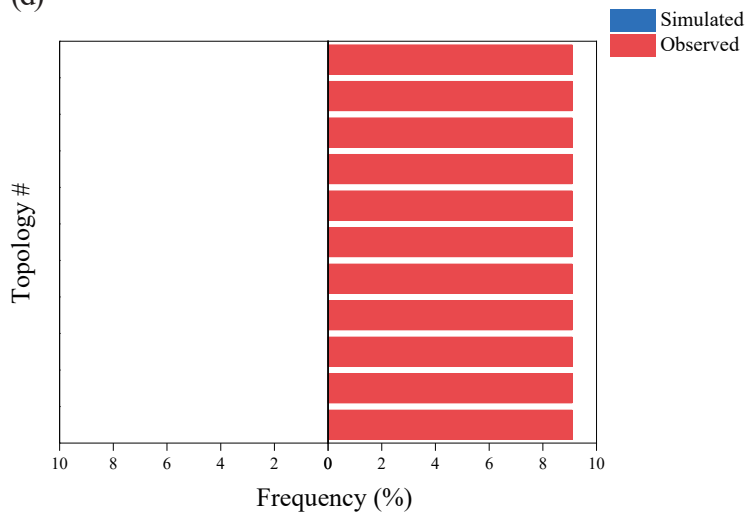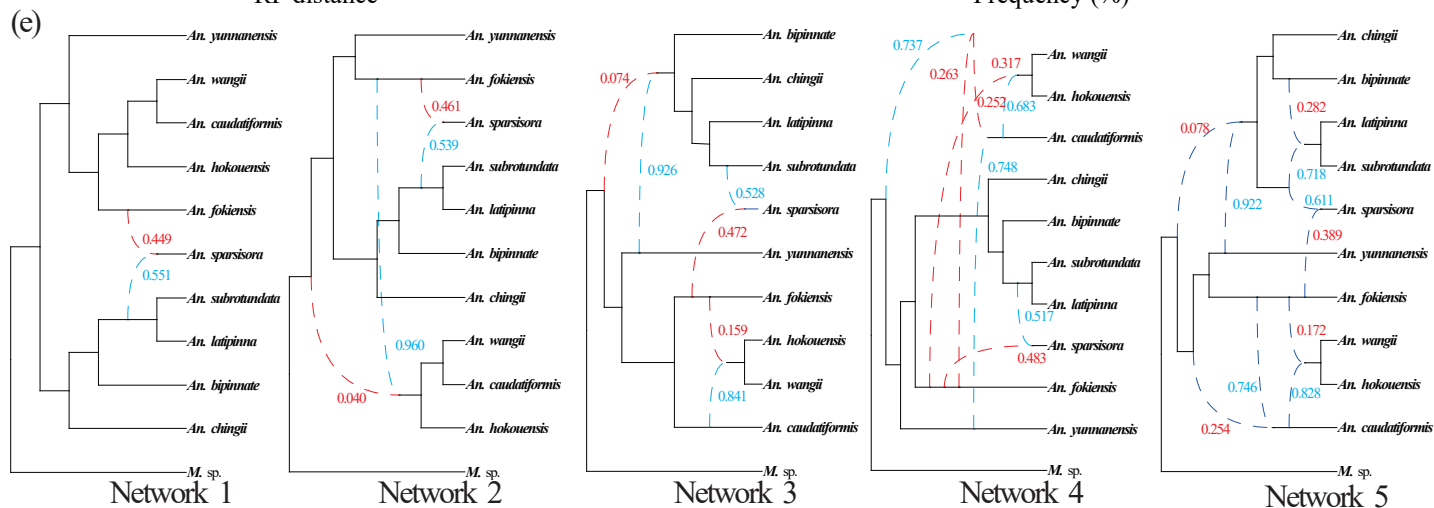

Supplement: Supplementary file 1 [file plants-12-02237-s001.zip › FigS10.pdf]

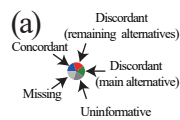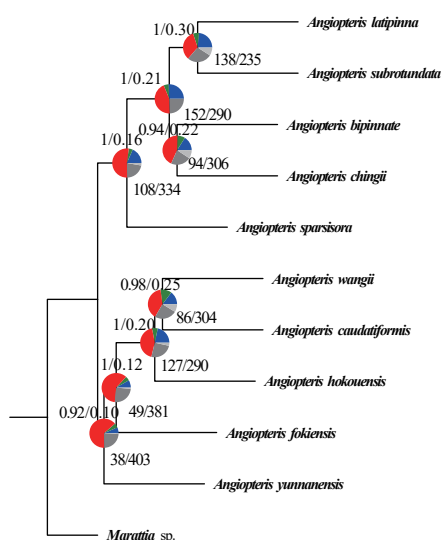

“*Archangiopteris*”  
“*Angiopteris* s.s.”

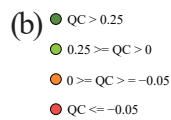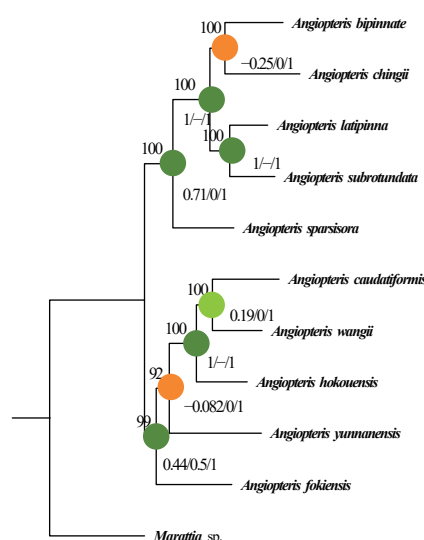

“*Archangiopteris*”  
“*Angiopteris* s.s.”

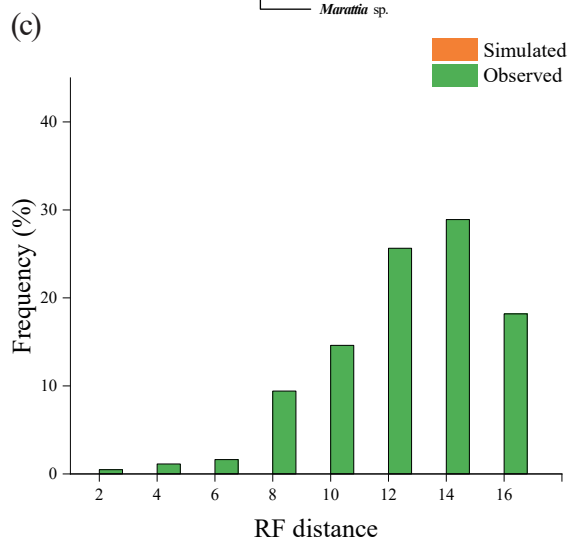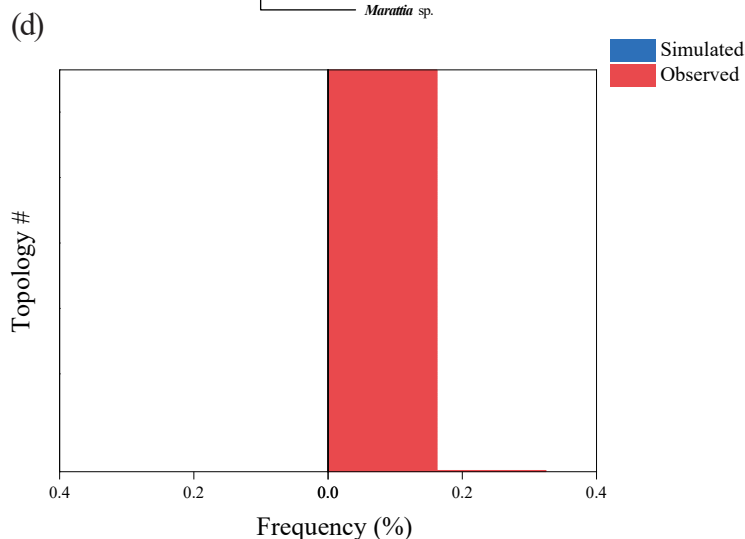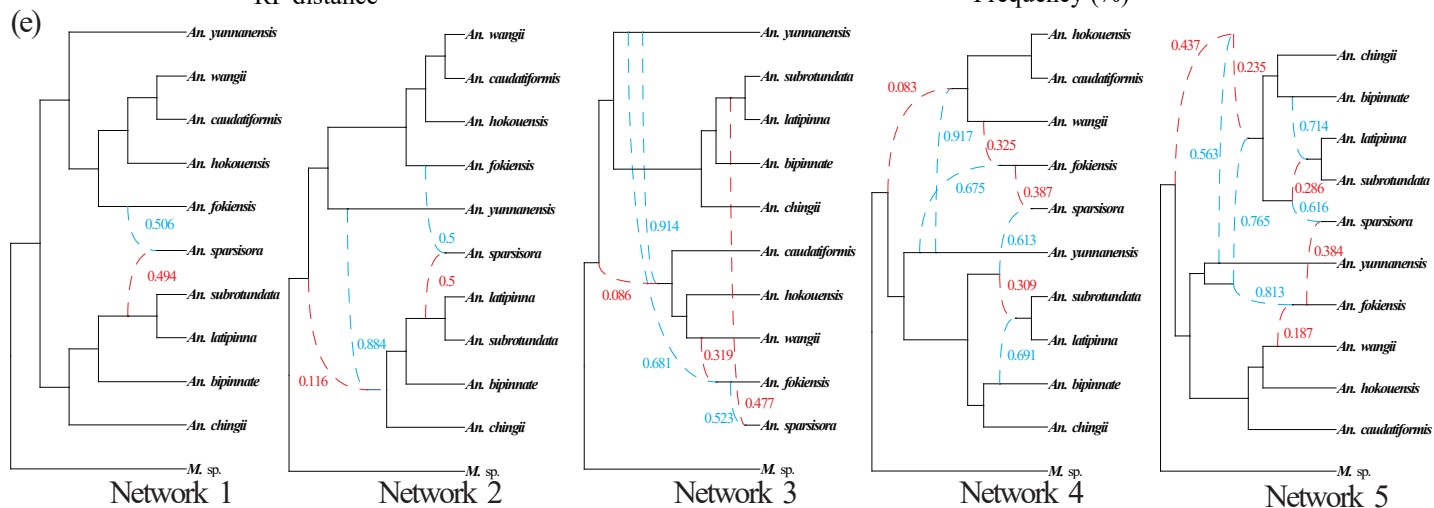

Supplement: Supplementary file 1 [file plants-12-02237-s001.zip › FigS11.pdf]

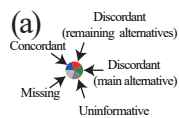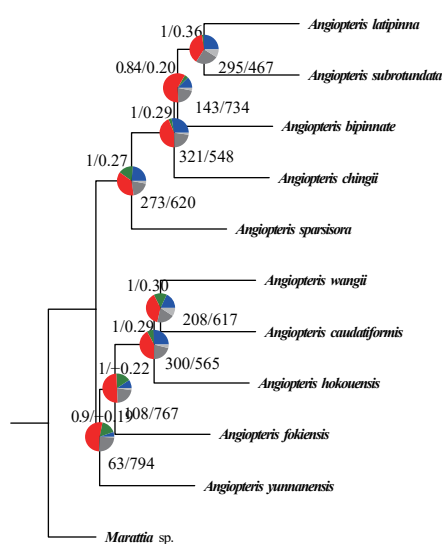

“*Archangiopteris*”

“*Angiopteris* s.s.”

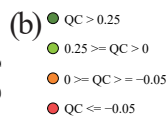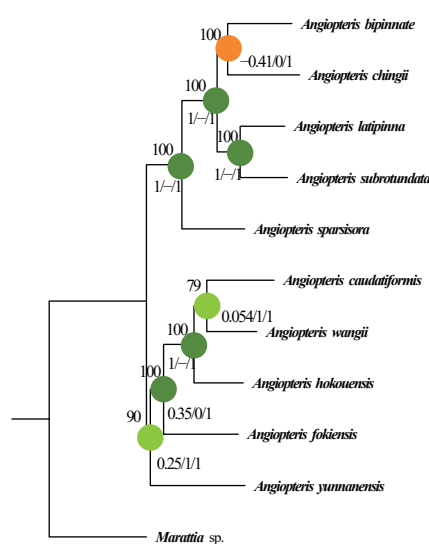

“*Archangiopteris*”

“*Angiopteris* s.s.”

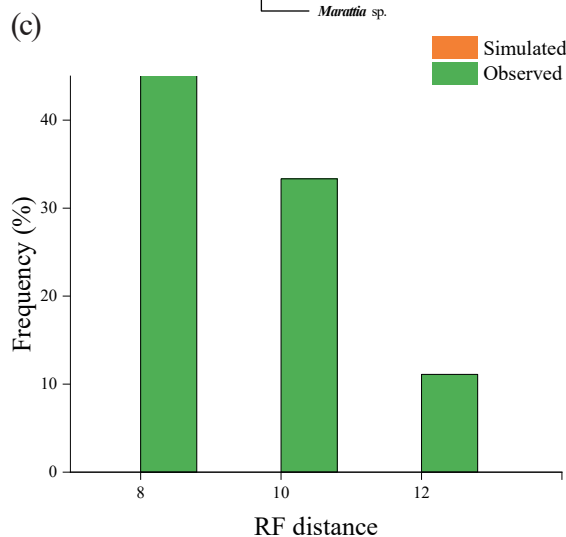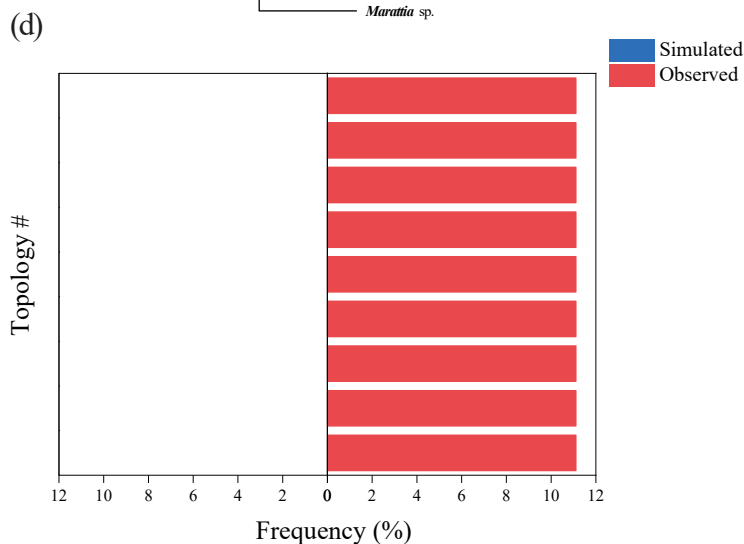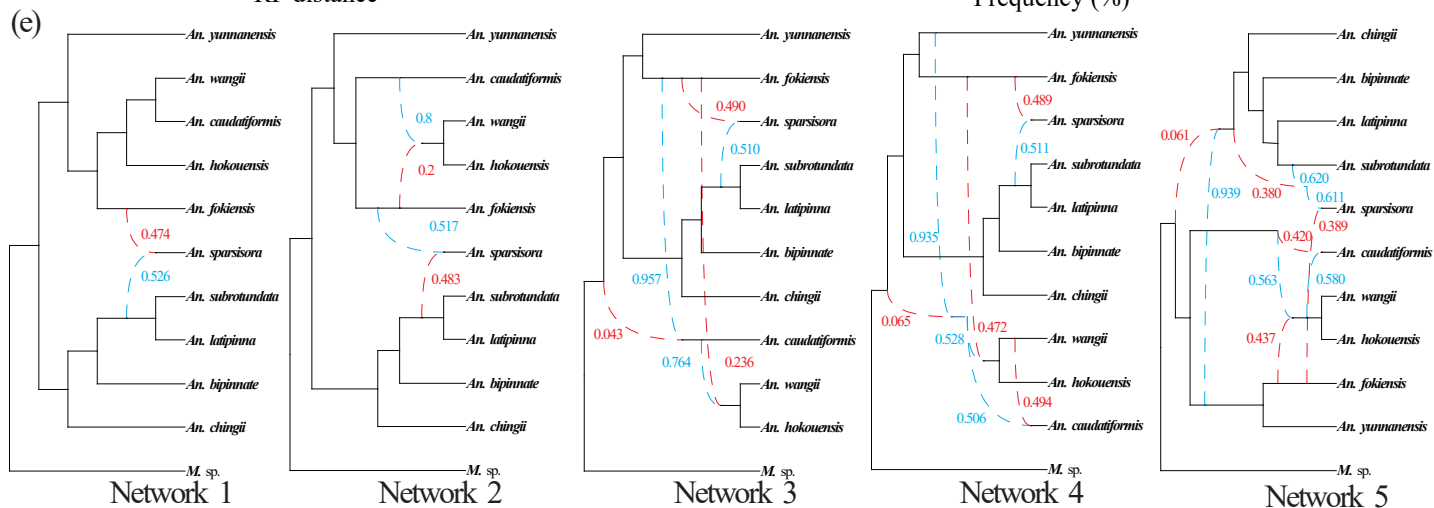

Supplement: Supplementary file 1 [file plants-12-02237-s001.zip › FigS12.pdf]

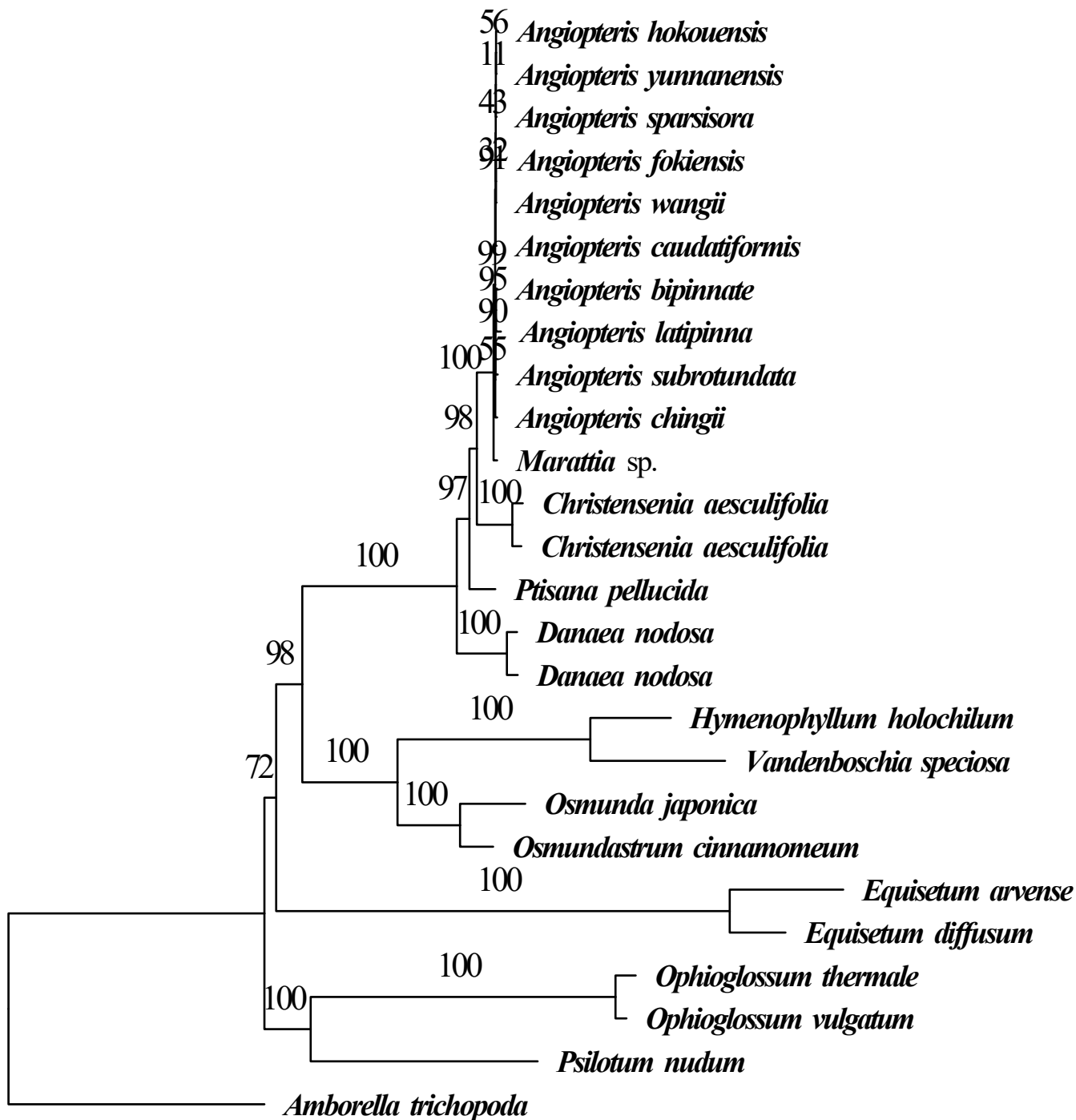

Supplement: Supplementary file 1 [file plants-12-02237-s001.zip › FigS13.pdf]

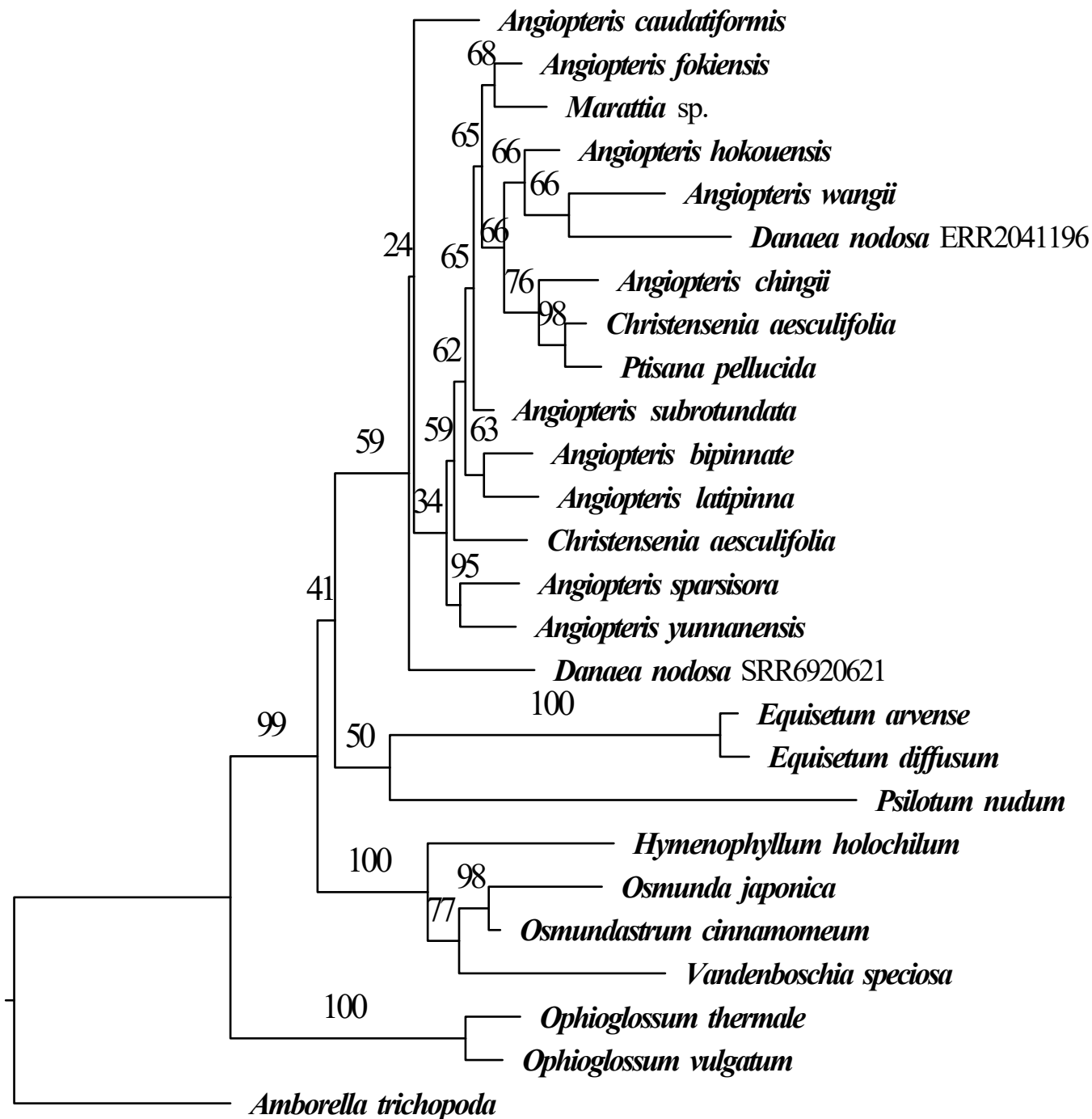

Supplement: Supplementary file 1 [file plants-12-02237-s001.zip › FigS14.pdf]

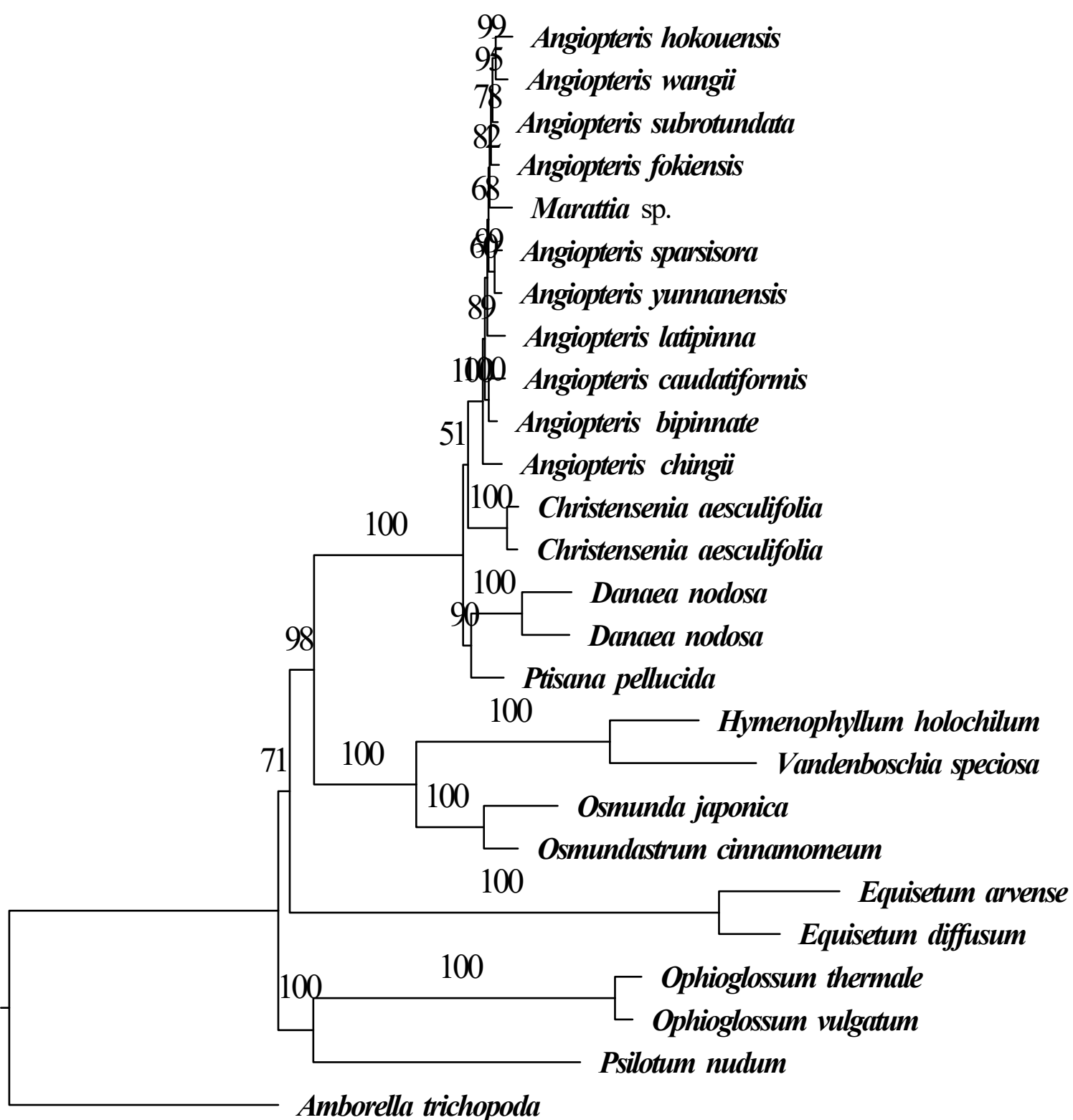

Supplement: Supplementary file 1 [file plants-12-02237-s001.zip › FigS15.pdf]

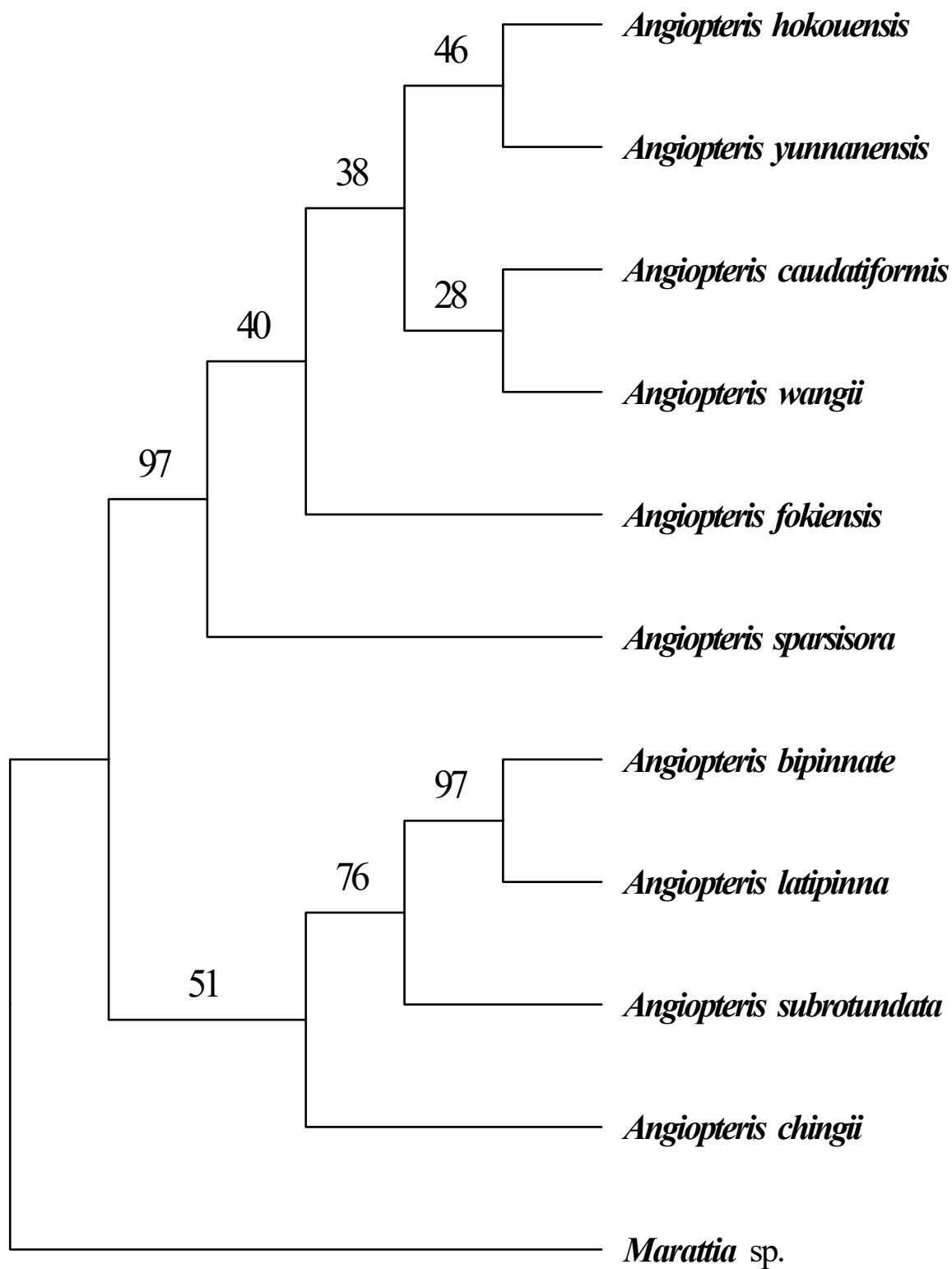

“*Angiopteris* s.s.”

“*Archangiopteris*”

Supplement: Supplementary file 1 [file plants-12-02237-s001.zip › FigS16.pdf]

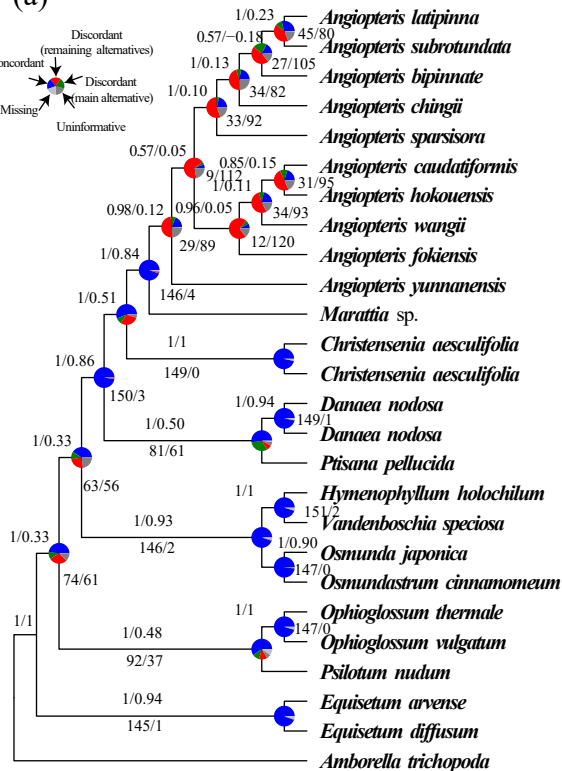

(b)

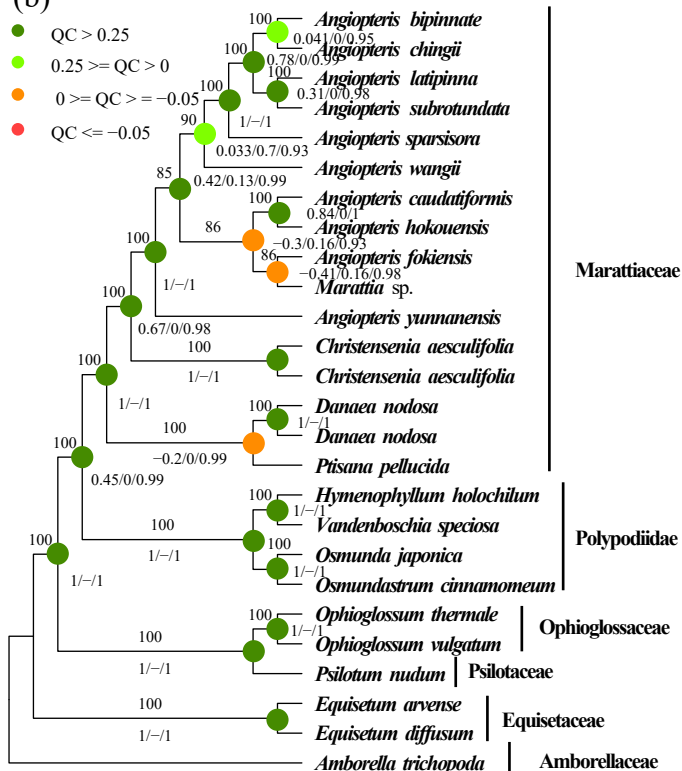

Supplement: Supplementary file 1 [file plants-12-02237-s001.zip › FigS2.pdf]

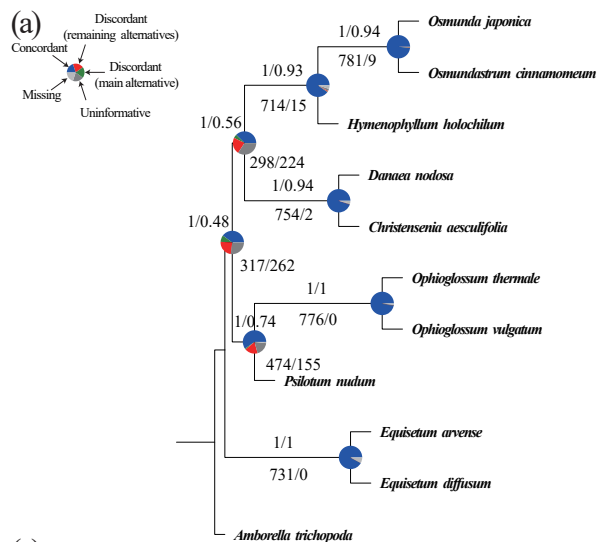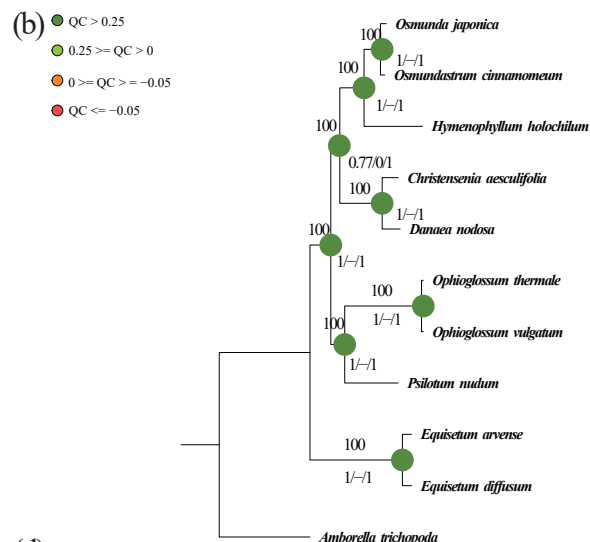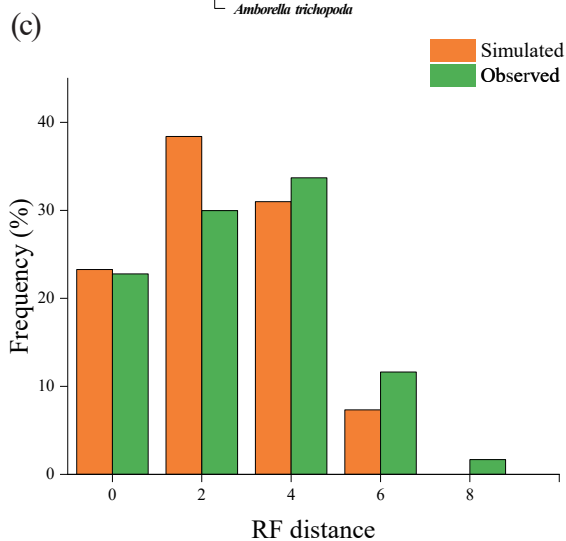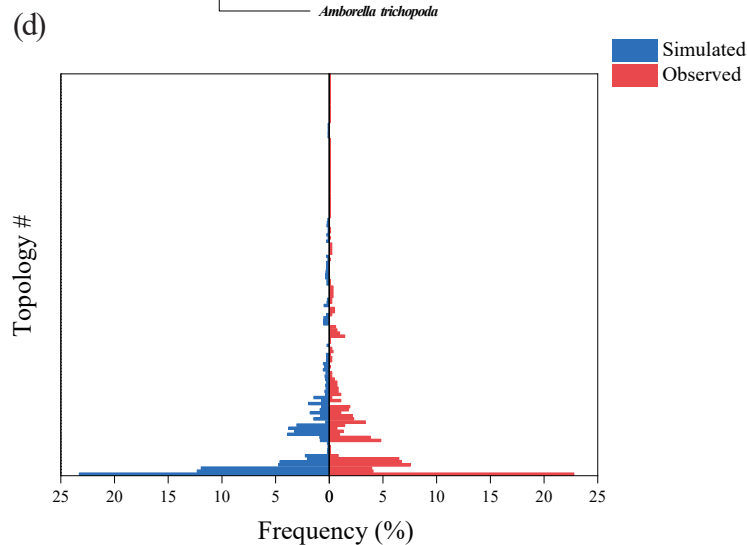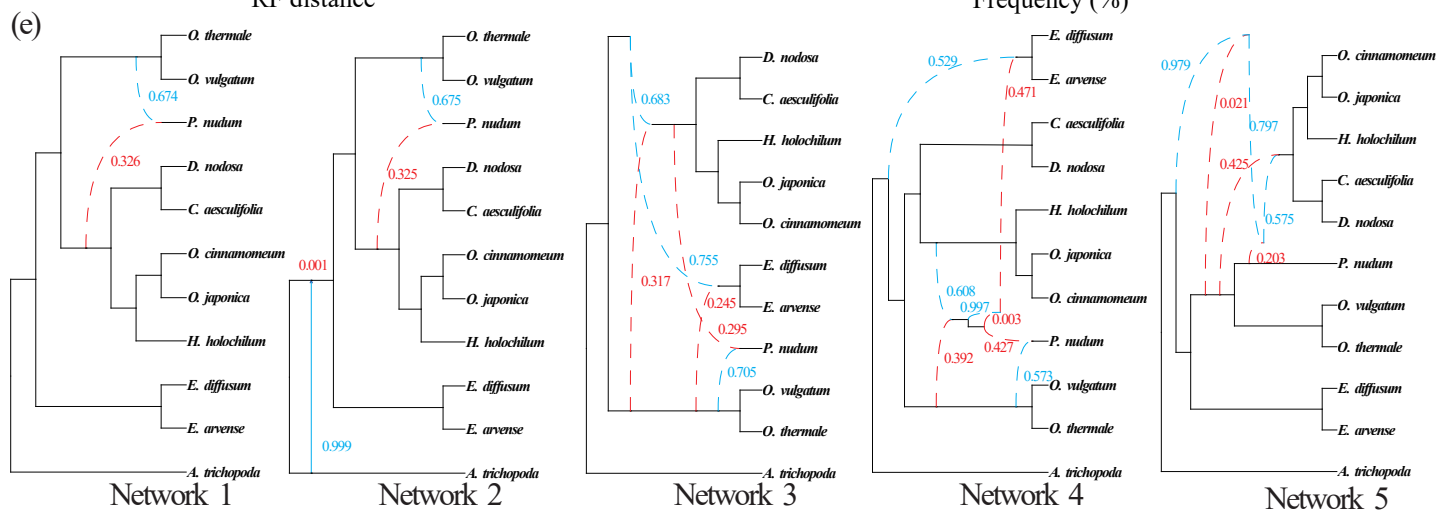

Supplement: Supplementary file 1 [file plants-12-02237-s001.zip › FigS3.pdf]

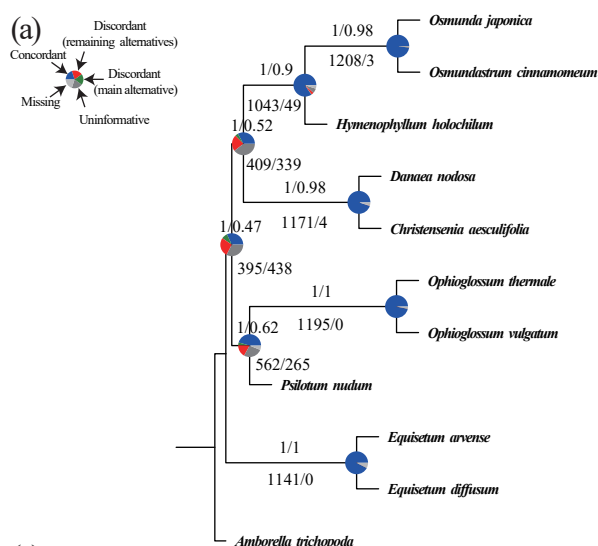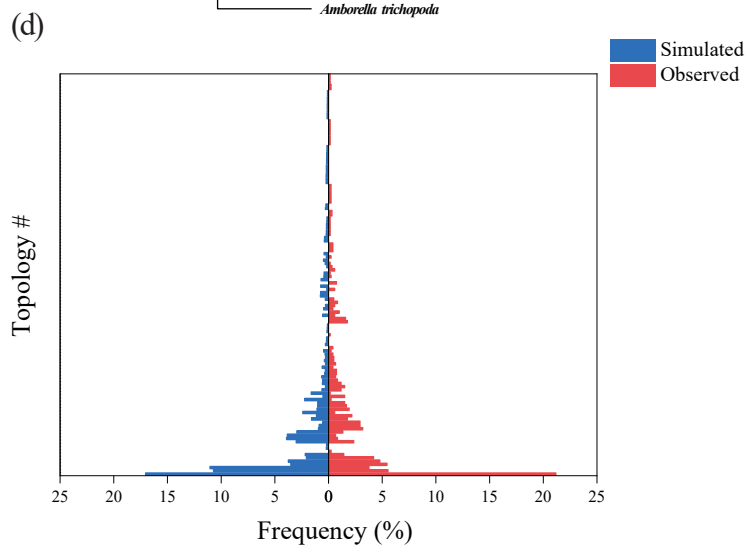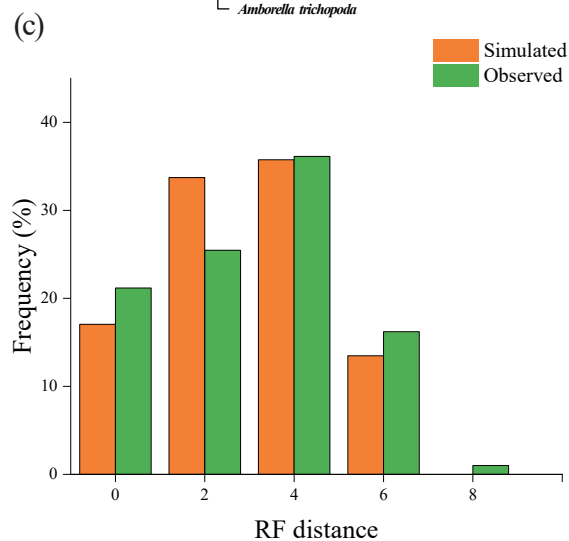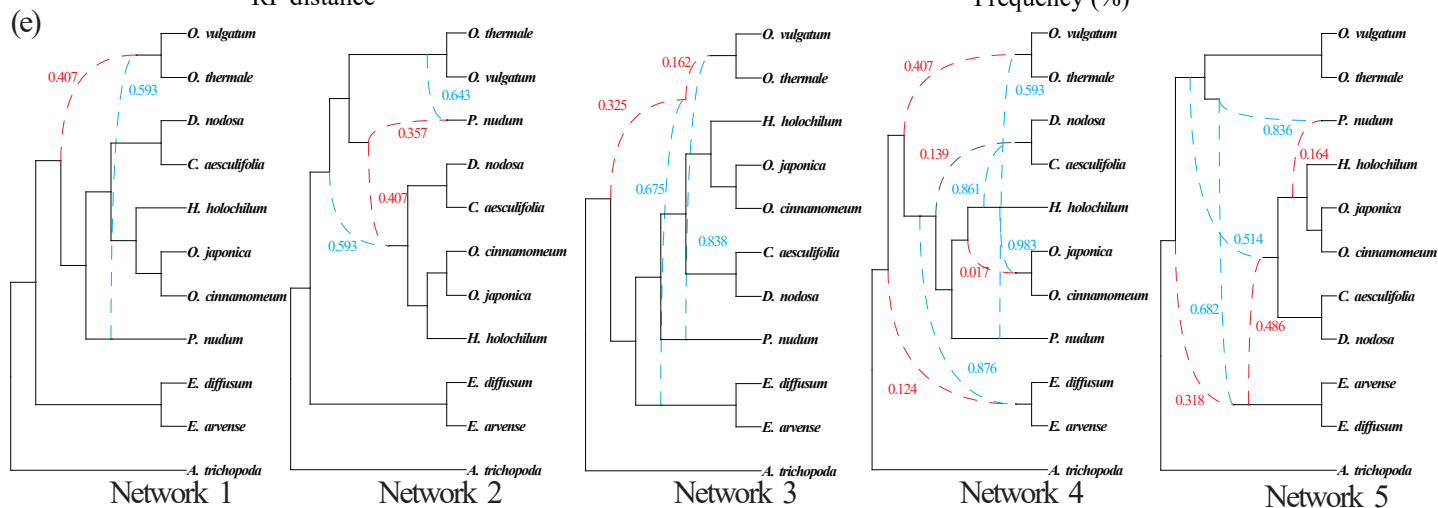

Supplement: Supplementary file 1 [file plants-12-02237-s001.zip › FigS4.pdf]

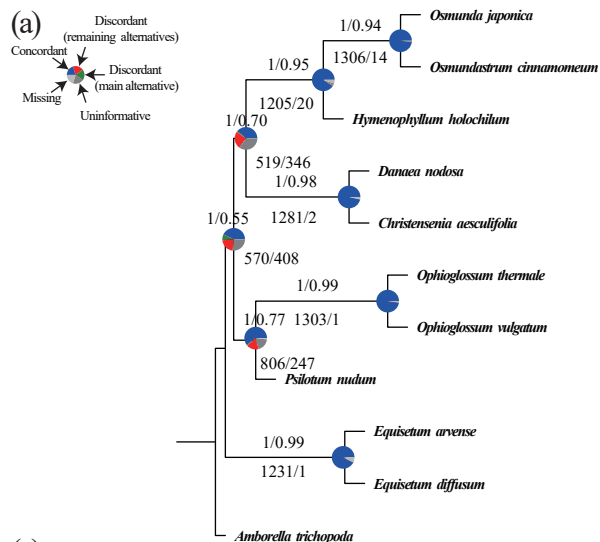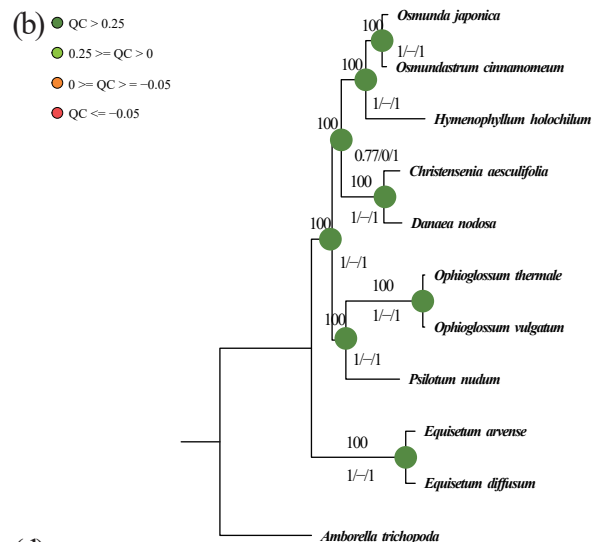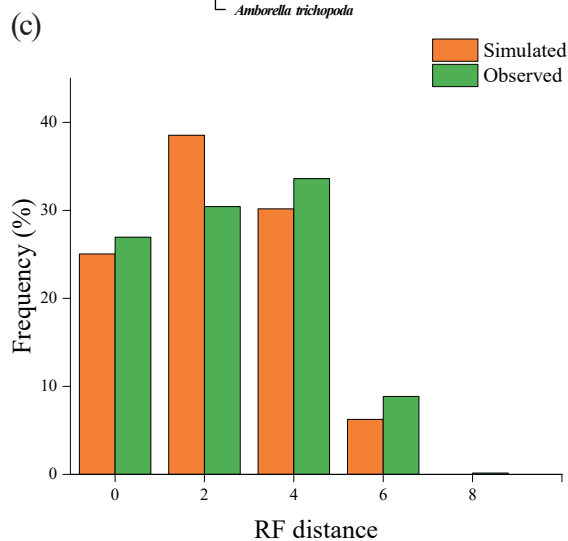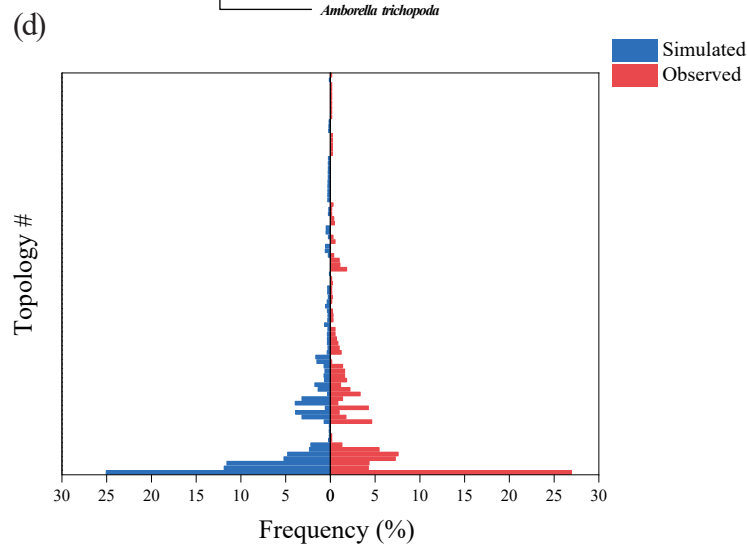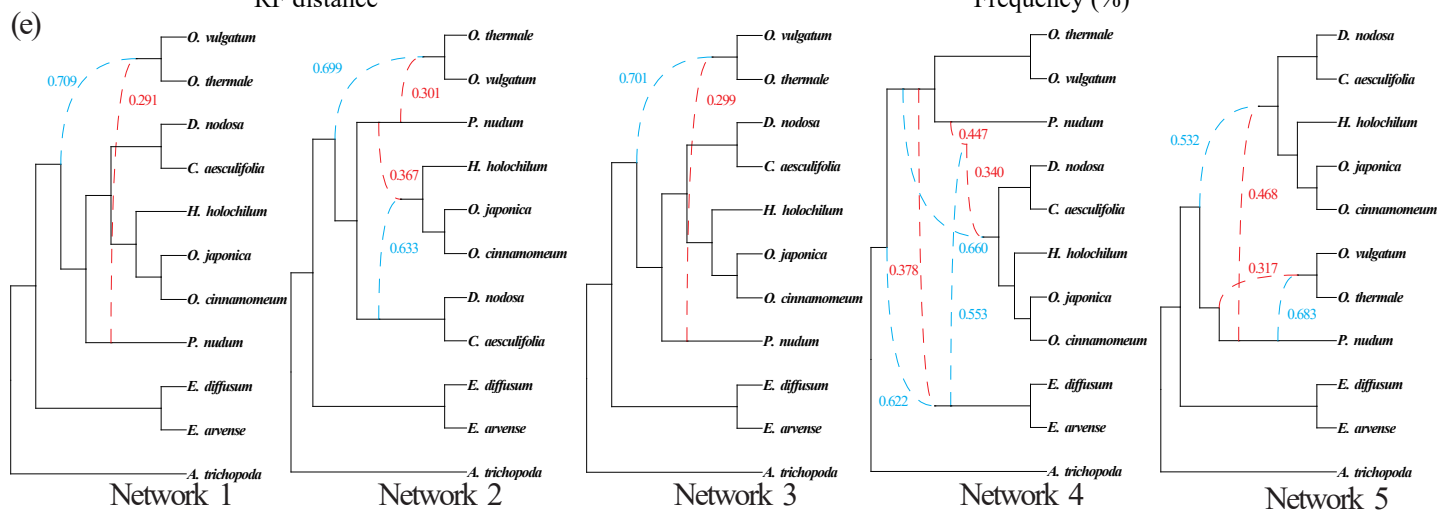

Supplement: Supplementary file 1 [file plants-12-02237-s001.zip › FigS5.pdf]

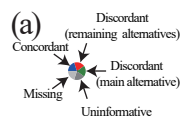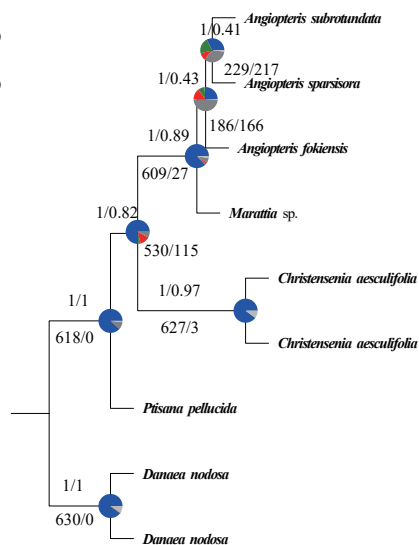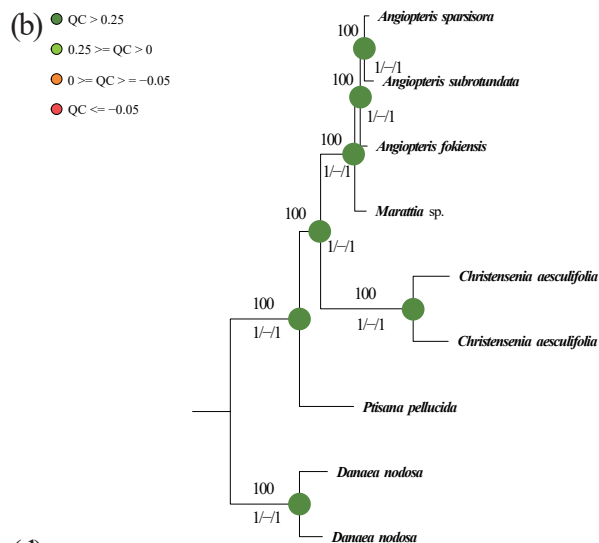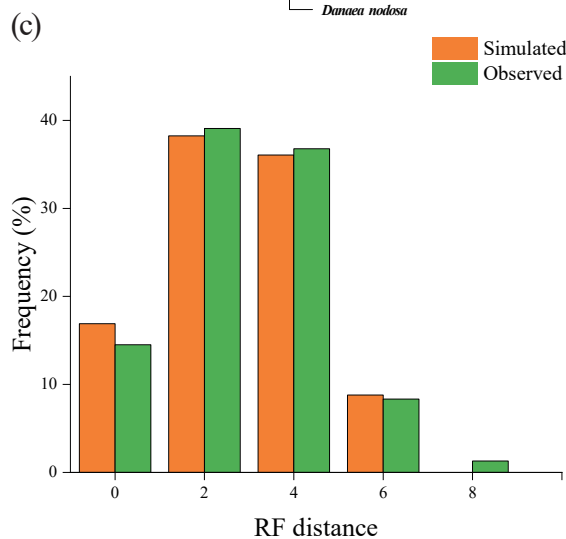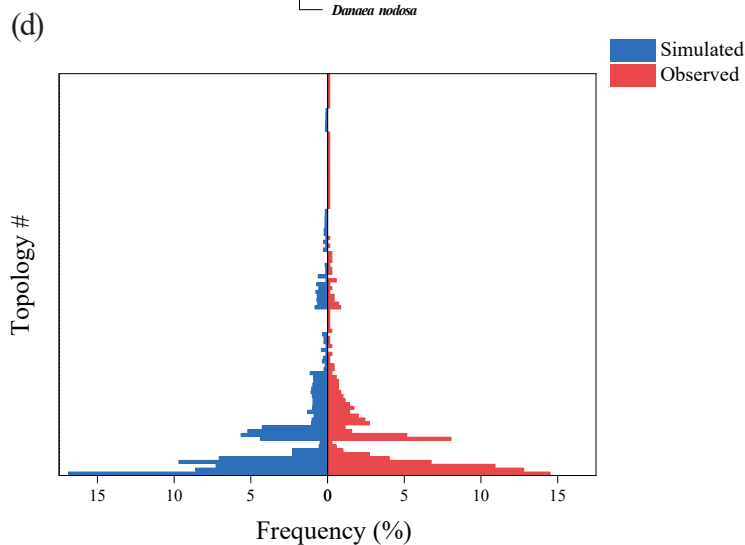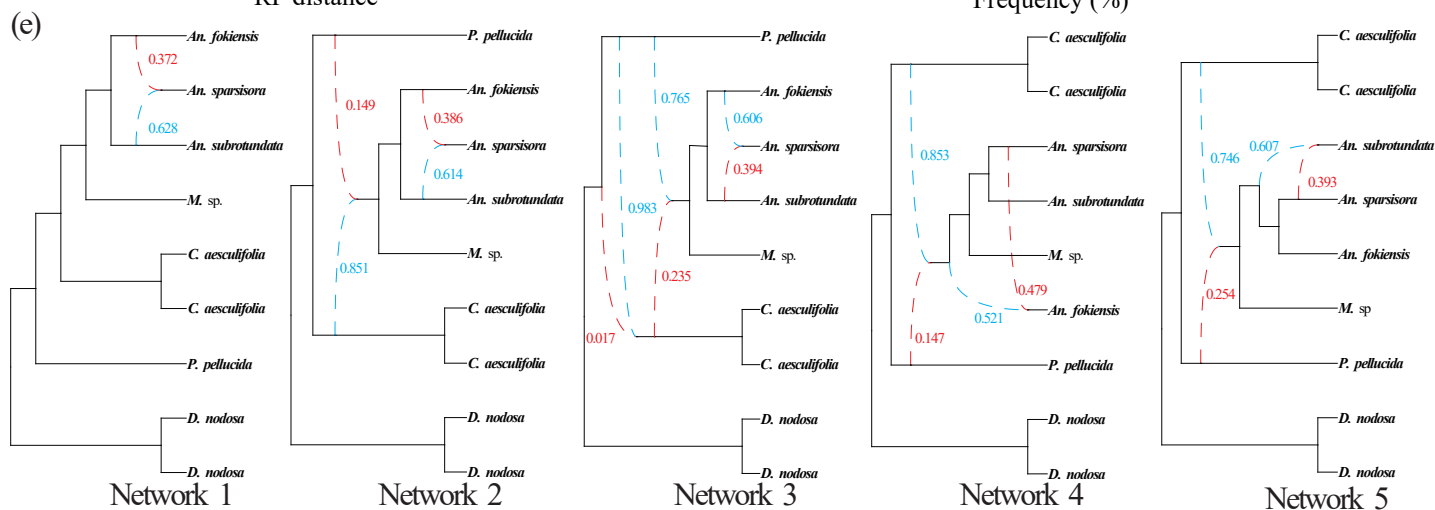

Supplement: Supplementary file 1 [file plants-12-02237-s001.zip › FigS7.pdf]

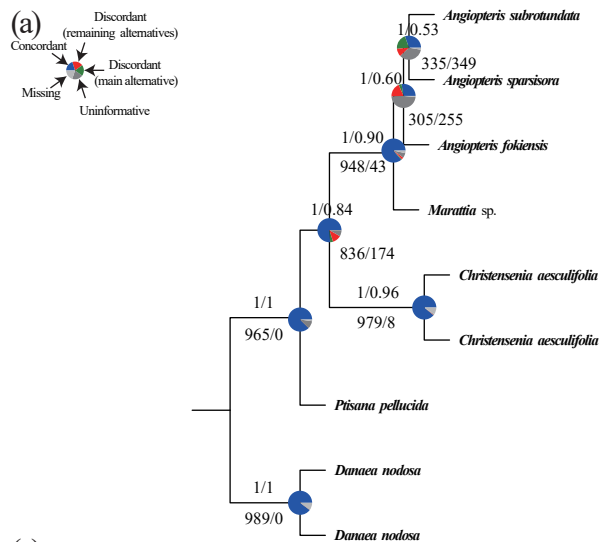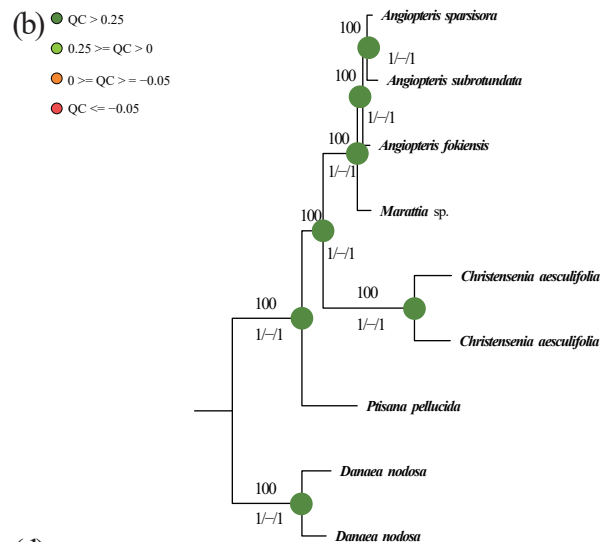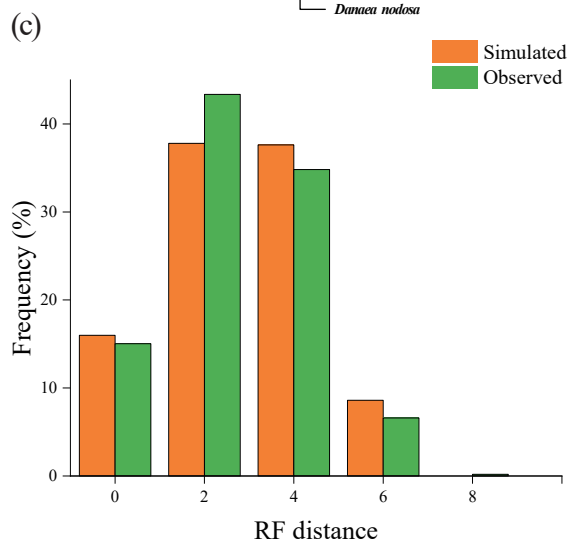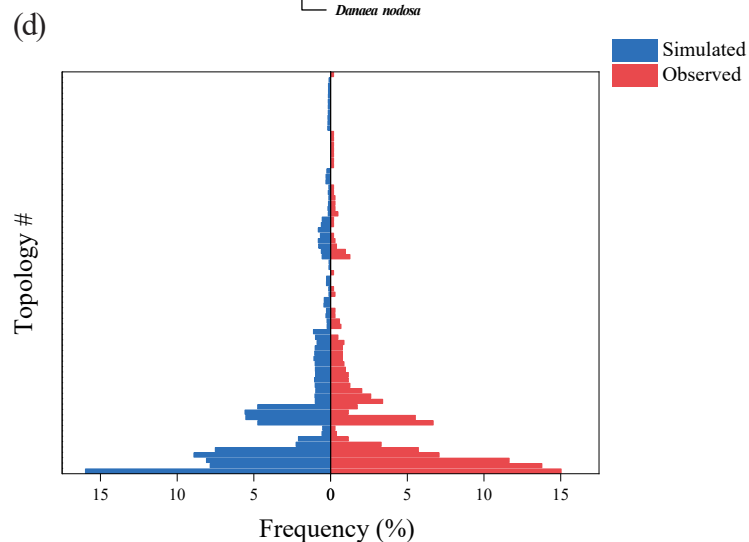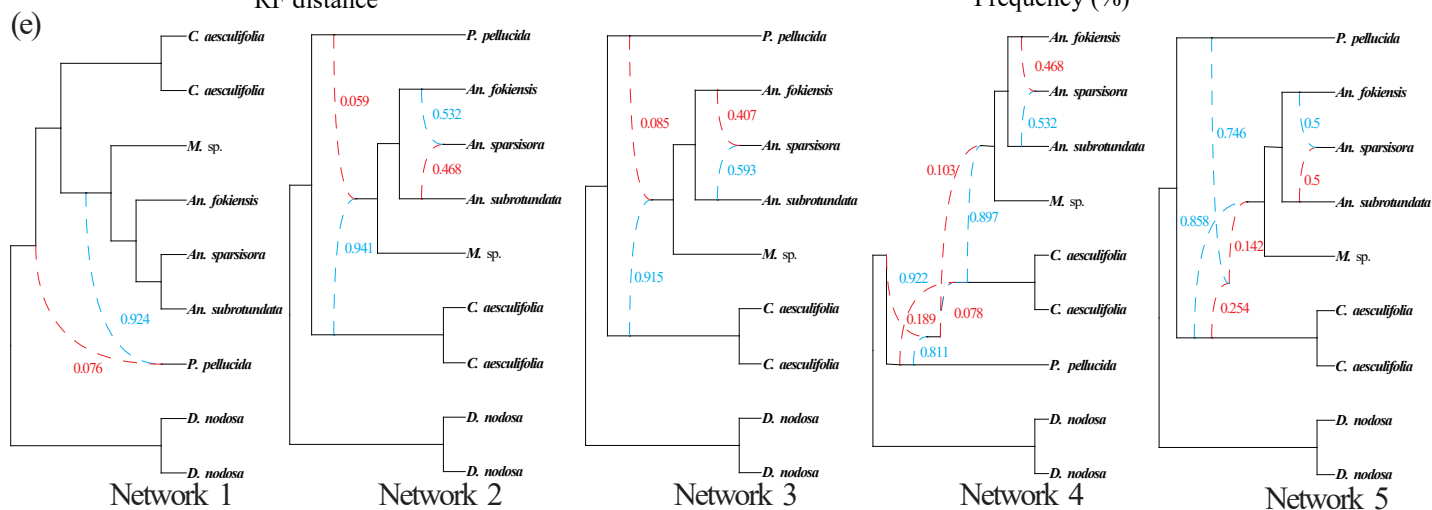

Supplement: Supplementary file 1 [file plants-12-02237-s001.zip › FigS8.pdf]

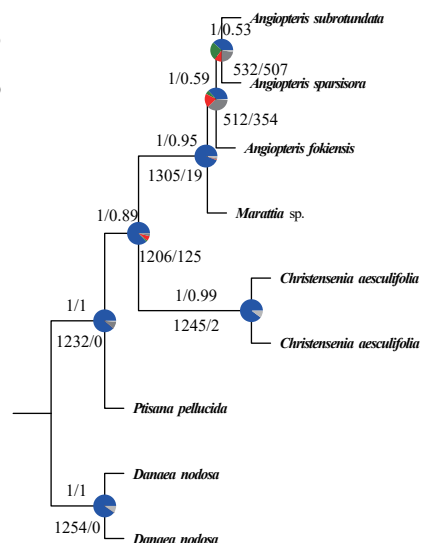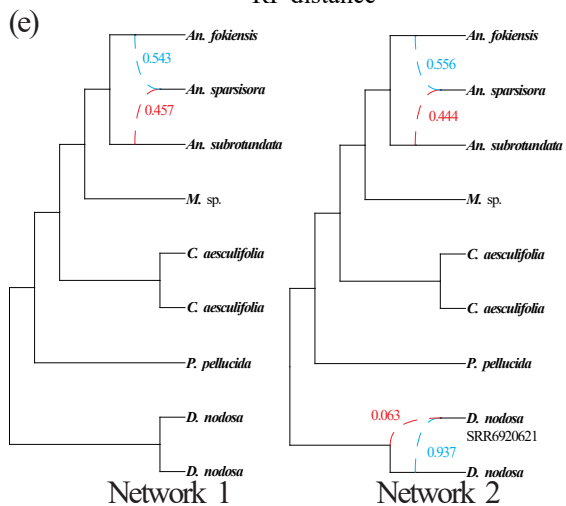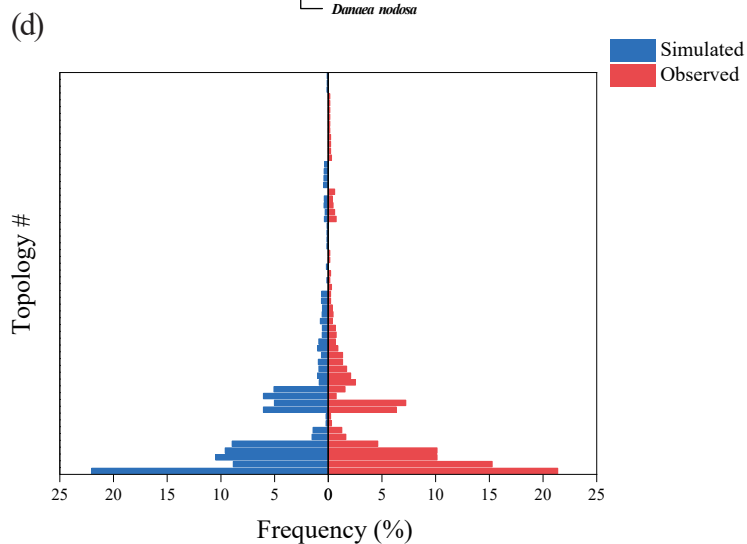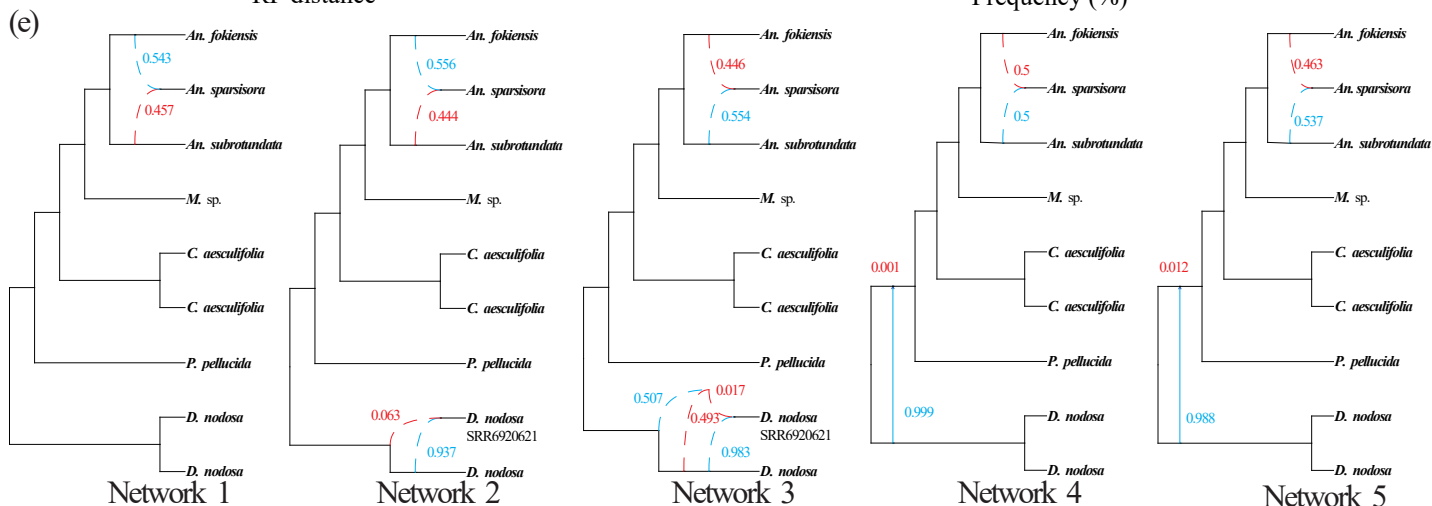

Supplement: Supplementary file 1 [file plants-12-02237-s001.zip › FigS9.pdf]
