# Supplementary material for: Transcriptome-Based Study on the Phylogeny and Hybridization of Marattialean Ferns (Marattiaceae)
Source: Plants (Basel). 2023 Jun 7;12(12):2237. doi: 10.3390/plants12122237 (PMC10301251; doi:10.3390/plants12122237)
Supplement: Supplementary file 1 [file FigS6.pdf › FigS6.pdf]

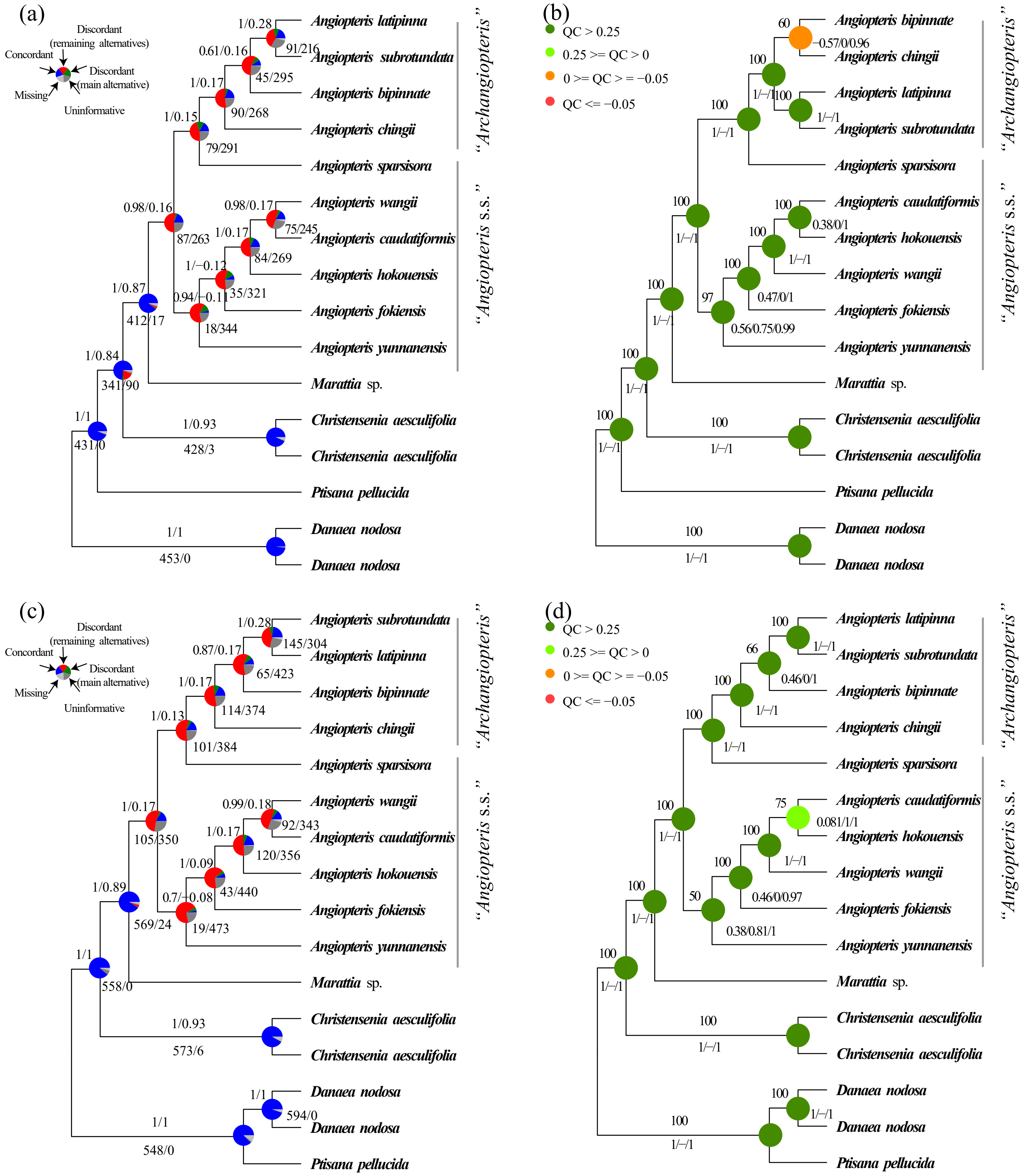

(a)
Discordant (remaining alternatives)
Concordant
Discordant (main alternative)
Missing
Uninformative
1/0.28
91/216
0.61/0.16
45/295
1/0.17
90/268
1/0.15
79/291
0.98/0.17
75/245
1/0.17
84/269
1/−0.12
35/321
0.94/−0.11
18/344
0.98/0.16
87/263
1/0.87
412/17
1/0.84
341/90
1/1
431/0
1/0.93
428/3
1/1
453/0
Angiopteris latipinna
Angiopteris subrotundata
Angiopteris bipinnate
Angiopteris chingii
Angiopteris sparsisora
Angiopteris wangii
Angiopteris caudatiformis
Angiopteris hokouensis
Angiopteris fokiensis
Angiopteris yunnanensis
Marattia sp.
Christensenia aesculifolia
Christensenia aesculifolia
Ptisana pellucida
Danaea nodosa
Danaea nodosa
"Archangiopteris"
"Angiopteris s.s."
(b)
QC > 0.25
0.25 >= QC > 0
0 >= QC > = −0.05
QC <= −0.05
60
−0.57/0/0.96
100
1/−/1
100
1/−/1
100
1/−/1
100
0.38/0/1
100
1/−/1
100
0.47/0/1
97
0.56/0.75/0.99
100
1/−/1
100
1/−/1
100
1/−/1
100
1/−/1
100
1/−/1
100
1/−/1
100
1/−/1
Angiopteris bipinnate
Angiopteris chingii
Angiopteris latipinna
Angiopteris subrotundata
Angiopteris sparsisora
Angiopteris caudatiformis
Angiopteris hokouensis
Angiopteris wangii
Angiopteris fokiensis
Angiopteris yunnanensis
Marattia sp.
Christensenia aesculifolia
Christensenia aesculifolia
Ptisana pellucida
Danaea nodosa
Danaea nodosa
"Archangiopteris"
"Angiopteris s.s."
(c)
Discordant (remaining alternatives)
Concordant
Discordant (main alternative)
Missing
Uninformative
1/0.28
145/304
0.87/0.17
65/423
1/0.17
114/374
1/0.13
101/384
0.99/0.18
92/343
1/0.17
120/356
1/0.09
43/440
0.7/−0.08
19/473
1/0.17
105/350
1/0.89
569/24
1/1
558/0
1/0.93
573/6
1/1
594/0
1/1
548/0
Angiopteris subrotundata
Angiopteris latipinna
Angiopteris bipinnate
Angiopteris chingii
Angiopteris sparsisora
Angiopteris wangii
Angiopteris caudatiformis
Angiopteris hokouensis
Angiopteris fokiensis
Angiopteris yunnanensis
Marattia sp.
Christensenia aesculifolia
Christensenia aesculifolia
Danaea nodosa
Danaea nodosa
Ptisana pellucida
"Archangiopteris"
"Angiopteris s.s."
(d)
QC > 0.25
0.25 >= QC > 0
0 >= QC > = −0.05
QC <= −0.05
100
1/−/1
66
0.46/0/1
100
1/−/1
100
1/−/1
75
0.081/1/1
100
1/−/1
100
0.46/0/0.97
50
0.38/0.81/1
100
1/−/1
100
1/−/1
100
1/−/1
100
1/−/1
100
1/−/1
100
1/−/1
Angiopteris latipinna
Angiopteris subrotundata
Angiopteris bipinnate
Angiopteris chingii
Angiopteris sparsisora
Angiopteris caudatiformis
Angiopteris hokouensis
Angiopteris wangii
Angiopteris fokiensis
Angiopteris yunnanensis
Marattia sp.
Christensenia aesculifolia
Christensenia aesculifolia
Danaea nodosa
Danaea nodosa
Ptisana pellucida
"Archangiopteris"
"Angiopteris s.s."
